# An integrated network toxicology and multi-omics framework prioritizes BRCA1 as a testable candidate in benzo[a]pyrene-associated oral cancer

**DOI:** 10.3389/fphar.2026.1940046

**Published:** 2026-09-15

**Authors:** Weijia Ye, Zhenyi Liu, Peng He

**Affiliations:** 1 Department of Stomatology, Lishui Central Hospital, Lishui, Zhejiang, China; 2 Medical school, Shandong Xiehe University, Jinan, Shandong, China

**Keywords:** benzo[a]pyrene (BaP), BRCA1, multi-omics, network toxicology, oral cancer

## Abstract

Environmental exposure to benzo[a]pyrene (BaP) is a recognized risk factor for oral cancer, but systematic strategies for prioritizing candidate molecular nodes and generating experimentally testable hypotheses remain limited. Here, we conducted a hypothesis-generating, methodologically oriented exploratory study using an integrated three-tier framework comprising computational target prioritization, multi-omics contextualization, and preliminary phenotypic assessment. Network toxicology identified four genes shared between the predefined BaP-associated and oral cancer-related gene sets: EGFR, HRAS, TP53, and BRCA1. These genes constituted the complete intersection and were not ordered by a composite score. BRCA1 was selected as a study-specific, testable candidate for focused follow-up based on convergent expression, protein-context, and DNA-damage-response evidence. Exploratory docking and molecular dynamics analyses characterized a predicted BaP–BRCA1 structural model but did not establish direct biochemical binding. Single-cell and spatial transcriptomic analyses described the baseline distribution of BRCA1 across tumor, immune, and stromal compartments but lacked BaP exposure annotations. Separately, BaP treatment was accompanied by increased proliferation and BRCA1 mRNA expression in CAL27 and SCC9 cells, whereas BRCA1 knockdown attenuated BaP-associated proliferation and was accompanied by changes in p53-axis transcript and protein readouts. This exploratory study prioritizes BRCA1 as a testable candidate and illustrates the utility of integrating network toxicology with multi-omics contextualization. The current findings do not establish direct BaP–BRCA1 binding, direct regulation of BRCA1 by BaP, a definitive *in vivo* mechanism, or clinical causality. All mechanistic and clinical interpretations require further validation through direct biochemical assays, additional experimental models, and exposure-annotated human cohorts.

## 1 Introduction

Oral cancer, predominantly oral squamous cell carcinoma (OSCC), remains a major global health burden, with advanced-stage disease often compromising therapeutic efficacy and long-term survival (Karunakaran and Muniyan, 2020; Mosaddad et al., 2021). Among its multifactorial etiology, environmental exposures constitute a critical and potentially modifiable risk category (Nokovitch et al., 2023). Benzo[a]pyrene (BaP), a prototypical polycyclic aromatic hydrocarbon classified as a Group I carcinogen (Saravanakumar et al., 2022; Huang et al., 2020), undergoes metabolic activation by cytochrome P450 enzymes to generate reactive intermediates, most notably BPDE, which form covalent DNA adducts and drive mutagenesis and malignant transformation (Bukowska et al., 2022; Pandelides et al., 2023; Park et al., 2017). Experimental evidence directly links BaP to oral cancer-relevant phenotypes (e.g., enhanced proliferation and pro-metastatic behavior in OSCC cells), and BaP-derived DNA adducts are detectable in human buccal mucosa; however, the specific molecular pathways mechanistically connecting BaP-associated genotoxic stress to oral cancer progression remain incompletely defined (He et al., 2022).

BRCA1 is well established for its canonical functions in safeguarding genome integrity, encompassing DNA damage signaling, homologous recombination repair, and cell cycle checkpoint regulation (Romagnolo et al., 2015; Tang et al., 2024; Harbi and Aschner, 2024). Intriguingly, in established malignancies, BRCA1 dysregulation—including context-dependent overexpression—has been increasingly linked to tumor progression and adverse outcomes, possibly reflecting a state of “non-oncogenic addiction” wherein cancer cells rely on BRCA1 to cope with persistent genotoxic stress (Gedeonová et al., 2025; Yu et al., 2024). BRCA1 functions as a repair pathway switch, governing the choice between homologous recombination and non-homologous end joining, and BaP may alter its affinity for partners such as PALB2, RAD51, or 53BP1 (Yun and Hiom, 2009; Bartke and Groth, 2019). Given that BaP bioactivation generates bulky DNA adducts and oxidative damage, BRCA1 emerges as a mechanistically plausible node linking exposure to DNA repair and checkpoint programs (Kang et al., 2011). Nevertheless, empirical evidence directly demonstrating specific binding or functional perturbation of BRCA1 by BaP in oral cancer remains scarce (He et al., 2022).

Traditional toxicological studies typically focus on a limited set of canonical pathways and often fail to capture the complexity of carcinogen-induced cellular perturbations at the molecular network level (Jin et al., 2022). Consequently, there is an urgent need for systems-level analytical strategies that systematically prioritize candidate mediators and generate experimentally testable hypotheses (Li et al., 2025). To address these gaps, we established an integrated three-tier framework comprising computational target prioritization, multi-omics contextualization, and preliminary phenotypic assessment. The predefined intersection of the BaP-associated target set and the retained oral cancer-related gene set yielded four candidates: EGFR, HRAS, TP53, and BRCA1. This intersection did not impose a composite quantitative hierarchy. BRCA1 was selected as a study-specific, testable candidate for focused follow-up based on convergent expression, protein-context, and DNA-damage-response evidence. We then used public single-cell and spatial datasets to characterize the baseline cellular and regional distribution of BRCA1 in oral cancer and performed initial cell experiments to assess whether BaP treatment, BRCA1 expression, and cellular phenotypes were experimentally associated under defined *in vitro* conditions. The study was designed to generate testable hypotheses and was not intended to establish direct BaP–BRCA1 binding, a definitive toxicological mechanism, or a clinical causal relationship.

## 2 Materials and methods

### 2.1 Network toxicology analysis of Benzo[a]pyrene (BaP)

The molecular structure of benzo[a]pyrene (BaP) was retrieved from PubChem (https://pubchem.ncbi.nlm.nih.gov/). Toxicological annotations, including predicted LD_50_ and physicochemical properties, were obtained from ProTox-II (https://tox.charite.de/). BaP-associated targets were collected from STITCH (http://stitch.embl.de/), SwissTargetPrediction (http://www.swisstargetprediction.ch/), and ChEMBL (https://www.ebi.ac.uk/chembl/), and standardized to official gene symbols. For CTD, the top 50 BaP-associated genes ranked by the available chemical–gene interaction evidence were retained as an additional high-confidence evidence layer. After integration and removal of duplicate entries, the BaP-associated target union contained 122 unique genes. Oral cancer-related genes were retrieved from GeneCards (https://www.genecards.org/), ranked by the GeneCards relevance score, and the 50 genes retained in the original analysis were used as the predefined disease-gene set. It should be noted that the GeneCards relevance score primarily reflects the volume of published research for a gene–disease association rather than mechanistic or exposure-specific relevance. Consequently, well-studied oncogenes may systematically obtain high scores across multiple cancer types, which can bias the candidate set toward highly published genes irrespective of their specific relationship to BaP-associated oral carcinogenesis. Candidate genes were defined by the intersection of the 122-gene BaP target union and the 50-gene disease set. This intersection contained EGFR, HRAS, TP53, and BRCA1. No additional CTD-count, STRING-degree, or composite-score threshold was applied to reduce a larger overlap set to these four genes.

For quantitative reporting, BaP-gene interaction records were re-extracted from the official CTD chemical-gene interaction bulk report generated on 30 July 2026. For each intersection gene, the total number of curated interaction records and the number of unique supporting PubMed references were calculated. STRING v12.0 (accessed 21 August 2026) was used to generate an induced network containing EGFR, HRAS, TP53, and BRCA1 for *Homo sapiens*, with no additional interactors and a required interaction score of 0.400. Only the experiments and curated-databases channels were active; text mining, co-expression, phylogenetic co-occurrence, gene neighborhood, and gene fusion were inactive. Restricted combined scores were calculated from the active channel scores according to the STRING probabilistic integration procedure. Node degree was defined as the number of retained connections of each gene within this four-node network.

### 2.2 Analysis of candidate target gene expression in tumors

To evaluate whether candidate targets exhibit differential expression in head and neck squamous cell carcinoma (HNSC), we used GEPIA2 (http://gepia2.cancer-pku.cn/) to compare TCGA tumor samples with GTEx normal tissues. Genes exhibiting statistically significant differential expression under GEPIA2 default settings were retained for downstream analysis, including BRCA1. Pan-cancer BRCA1 expression across 33 tumor types and corresponding normal tissues was queried using TIMER (http://timer.cistrome.org/). Immunohistochemical staining images and summaries for BRCA1 in HNSC and normal oral tissues were obtained from the Human Protein Atlas (HPA) (https://www.proteinatlas.org/).

### 2.3 Molecular docking

The amino acid sequence of BRCA1 was obtained from UniProt (https://www.uniprot.org/). A three-dimensional (3D) structure of the BRCA1 tandem BRCT domain was obtained from the RCSB Protein Data Bank (PDB ID: 3K16) and used for both docking and subsequent MD simulations. Protein and ligand structures were prepared by removing water molecules and adding hydrogen atoms using AutoDockTools/AutoDock 4. Protein and ligand structures were prepared by removing water molecules and adding hydrogen atoms using AutoDockTools/AutoDock 4 (https://autodock.scripps.edu/download-autodock4). Docking was performed with AutoDock Vina 1.2.5 (https://github.com/ccsb-scripps/AutoDock-Vina/releases). Docking was employed solely to assess whether BaP could adopt an energetically favorable and structurally plausible pose in the modeled BRCA1 system *in silico*. No direct biochemical binding or cellular target-engagement assays, including surface plasmon resonance (SPR) or cellular thermal shift assay (CETSA), were performed in this study. Therefore, the docking results were interpreted as hypothesis-generating evidence of structural plausibility rather than biochemical proof of a direct BaP–BRCA1 interaction. Docking poses and predicted interactions were visualized using PyMOL 3.1.

### 2.4 Analysis of BRCA1 in normal tissues

BRCA1 expression across normal tissues and corresponding single-cell summaries were obtained from HPA. Subcellular localization information was retrieved from ExMdb (http://www.bio-server.cn/ExMdb/). BRCA1 chromosomal localization was confirmed using the UCSC Genome Browser (https://genome.ucsc.edu/).

### 2.5 Single-cell and spatial transcriptomic analysis of BRCA1

Single-cell and spatial transcriptomic analyses were performed using OralDB (https://compbio.cn/oraldb/), which curate oral cancer–related scRNA-seq and spatial transcriptomic resources. For scRNA-seq, GEO datasets GSE173468, GSE181919, and GSE234933 were used. For spatial transcriptomics, GEO dataset GSE208253 was analyzed, and three patient samples were selected for visualization and comparative assessment. Dataset selection criteria (added for transparency): datasets were included based on the following predefined criteria: (i) human oral squamous cell carcinoma–related samples; (ii) availability of expression matrices enabling BRCA1 quantification; (iii) availability of cell-type annotations or metadata supporting compartment-level interpretation; (iv) adequate sequencing depth and platform quality to support robust visualization; and (v) sufficient sample size and quality for downstream analyses. Because BaP exposure annotation was not available for these public datasets, single-cell and spatial analyses were used to map the cellular and spatial context of BRCA1 in oral cancer rather than to infer BaP-driven microenvironmental dynamics *in vivo*. It should be emphasized that, as the public single-cell and spatial transcriptomic datasets lack BaP exposure annotation, the following analyses serve solely to characterize the cellular and spatial distribution features of BRCA1 in oral cancer, and do not infer BaP-driven *in vivo* microenvironmental dynamics.

### 2.6 Molecular functional analysis of BRCA1

Protein-association partners of BRCA1 were obtained from STRING (https://string-db.org/) and BioGRID (https://thebiogrid.org/). For Figure 5A, STRING v12.0 (accessed 21 August 2026) was queried for H. sapiens using BRCA1 as the query protein, a required interaction score of 0.400, 10 first-shell partners, and no second-shell partners. Partner ranking and edge retention were based on combined scores calculated with only the experiments and curated-databases channels active; text mining, co-expression, phylogenetic co-occurrence, gene neighborhood, and gene fusion were inactive. The resulting STRING edges were treated as functional protein associations supported by the selected channels and not necessarily as direct binary physical interactions. For Figure 5B, the BioGRID v5.0.260 multi-validated physical-interaction dataset was filtered to retain BRCA1-anchored human-human records and exclude BRCA1 self-interactions. The 20 displayed partners were ranked by the number of unique supporting PubMed references, followed by the number of experimental systems, record count, and gene symbol. Channel-level STRING scores and BioGRID experimental systems and PubMed references are reported in Supplementary Table S3. TCGA-HNSC gene expression data were downloaded via UCSC Xena (https://xenabrowser.net/datapages/). Samples were stratified into BRCA1-high and BRCA1-low groups using the median BRCA1 expression. Differentially expressed genes between groups were identified, and functional enrichment analyses were conducted using the R package clusterProfiler. Gene Ontology (GO) and KEGG pathway enrichment were performed, and multiple testing was adjusted using the Benjamini-Hochberg method, with adjusted P < 0.05 considered significant. Gene set enrichment analysis (GSEA) was performed on ranked gene lists to identify coordinated BRCA1-associated programs in HNSC.

### 2.7 Molecular dynamics (MD) simulation

To evaluate the stability of the predicted BRCA1–BaP association *in silico*, molecular docking and MD simulations were performed. The BRCA1 structure was obtained from the RCSB Protein Data Bank (https://www.rcsb.org/; PDB ID: 3K16). BaP was downloaded from PubChem in SDF format and converted to PDBQT using AutoDockTools 1.5.7 (https://autodock.scripps.edu/). Protein preparation included removal of water molecules, addition of polar hydrogens, and assignment of Kollman charges. Docking was performed using AutoDock Vina 1.2.5 with default parameters; the grid box encompassed the putative ligand-binding region. The top-ranked pose was used to initialize MD simulations. MD simulations were conducted in GROMACS 2022.1 (https://www.gromacs.org/) using the CHARMM36 force field for the protein. Ligand parameters were generated using the CGenFF server. The BRCA1–BaP complex was solvated in a triclinic TIP3P water box and neutralized with counterions. Systems underwent energy minimization, followed by equilibration under NVT and NPT ensembles. A 100 ns production run was performed at 300 K and 1 atm with periodic boundary conditions. Trajectory analyses included RMSD, RMSF, radius of gyration (Rg), and hydrogen-bond occupancy to evaluate complex stability. Trajectory analyses were performed using GROMACS. One independent 100-ns production trajectory was generated for each system (BRCA1–BaP and BRCA1–co-crystallized phosphopeptide). No additional independent MD replicas were performed. To assess within-trajectory variability, each trajectory was divided into five contiguous 20-ns blocks, and block-averaged RMSD and Rg values were calculated. These block metrics are reported as descriptive consistency indicators and are not interpreted as replicate-based standard errors. The uncertainty estimates for interaction energies (Err.Est.) were obtained via block averaging using gmx energy; they represent the uncertainty in the trajectory mean and are not standard deviations among independent replicas. Backbone RMSD values were calculated at 10 ps intervals across the 100 ns production trajectory and are reported as the mean ± temporal standard deviation. The 95th percentile was used as a descriptive upper bound. Statistics were calculated for the complete trajectory and for the final 20 ns interval. Per-residue RMSF was calculated across the complete production trajectory. Principal contact residues were operationally defined as residues located within 4.0 Å of BaP in at least three of five representative snapshots extracted at 0, 25, 50, 75, and 100 ns. Short-range protein–ligand interaction energies were extracted using the GROMACS energy module. The reported components included Coul-SR and LJ-SR terms. Mean values and temporal standard deviations were calculated across 10,001 frames. These terms represent short-range nonbonded interaction energies and do not include polar solvation, nonpolar solvation, or entropic contributions; they were therefore not interpreted as MM-PBSA or thermodynamic binding free energies. Molecular graphics and interactions were visualized using PyMOL 3.1. All docking and MD analyses were computational and were designed to characterize the structural plausibility and dynamic behavior of the predicted BRCA1–BaP model. No direct biochemical binding or cellular target-engagement assays, such as SPR or CETSA, were performed. Accordingly, persistence of the predicted configuration during simulation was not interpreted as experimental confirmation of direct BaP–BRCA1 binding.

To assess run-to-run variability, two additional independent 100-ns production simulations were performed for the BRCA1–BaP complex, resulting in three independent trajectories and a cumulative production time of 300 ns. All simulations used the same starting complex, force-field parameters, solvation conditions, equilibration procedure, temperature, and pressure settings, but independent initial atomic velocities were assigned using different random seeds. Backbone RMSD, radius of gyration (Rg), intermolecular hydrogen-bond number, and per-residue RMSF were calculated separately for each trajectory using identical analysis settings. For scalar trajectory metrics, a time-averaged value was first calculated for each independent run over the over the complete 100-ns production interval, after which the mean and standard deviation across the three independent runs were calculated. Per-residue RMSF variability was summarized as the mean ± standard deviation across the three trajectories.

### 2.8 Cell culture

The human oral squamous cell carcinoma cell line CAL27 (CL-0265) was obtained from Procell (Wuhan, China). Cells were cultured in DMEM/F-12 (1:1) (Gibco, USA) supplemented with 10% fetal bovine serum (ExCell Bio, Uruguay) and 1% penicillin–streptomycin (Gibco, USA) at 37 °C in a humidified atmosphere containing 5% CO_2_. Medium was refreshed every 2 days, and cells were passaged at 70%–80% confluence.

### 2.9 siRNA transfection

For BRCA1 knockdown, CAL27 cells were transfected with BRCA1-specific siRNA or negative control siRNA (GenePharma, Shanghai, China) using Lipofectamine 3000 (Invitrogen, USA) in Opti-MEM (Gibco, USA) according to the manufacturer’s protocol. Cells were seeded at 30%–40% confluence and transfected with 50 nM siRNA. After 24 h, cells were harvested or subjected to downstream treatments. Knockdown efficiency was confirmed by qRT-PCR.

### 2.10 Cell proliferation assay (CCK-8) and BaP treatment concentration

To assess proliferation following BaP exposure, 5,000 CAL27 cells per well were seeded in 96-well plates. After adhesion, cells were treated with BaP at 0, 0.1, 1, and 10 µM for 0, 12, 24, 36, and 48 h. At each time point, 10 µL CCK-8 reagent (Beyotime, China) was added and incubated for 1 h at 37 °C. Absorbance at 450 nm was measured using a microplate reader (Thermo Fisher, USA). All experiments were performed in triplicate. The 1 µM BaP concentration was not intended to represent an IC_50_; instead, it was selected as a sub-cytotoxic, biologically responsive dose to support mechanistic interrogation under our experimental conditions. The dose range (0.1–10 µM) was used to capture potential concentration-dependent effects.

### 2.11 Quantitative real-time PCR (qRT-PCR) and Western blotting

Total RNA was extracted using TRIzol (Takara, Japan). cDNA was synthesized with the Evo M-MLV RT Mix Kit (Accurate Biology, China) following the manufacturer’s instructions. qRT-PCR was performed on a LightCycler 480 II system (Roche, Switzerland) using SYBR Green Premix Pro Taq HS (Accurate Biology, China). Primers for BRCA1, CDKN1A, BAX, and GADD45A were synthesized by General Biosystems (Anhui, China), with β-actin as the internal control. Relative expression was calculated using the 2^−ΔΔCt method. Clinical trial number: not applicable.

Following the indicated treatments, CAL27 and SCC-9 cells were washed with ice-cold phosphate-buffered saline and lysed in RIPA buffer supplemented with protease inhibitors (Thermo Fisher Scientific). Protein concentrations were determined using a BCA protein assay kit (Thermo Fisher Scientific). Equal amounts of total protein (5 μg per lane) were separated by 10% SDS–PAGE and transferred onto PVDF membranes.The membranes were blocked with 5% nonfat milk in Tris-buffered saline containing 0.1% Tween-20 for 1 h at room temperature and incubated overnight at 4 °C with primary antibodies against CDKN1A/p21, GADD45A, BAX, and β-actin. After washing, the membranes were incubated with the corresponding HRP-conjugated secondary antibodies for 1 h at room temperature. All primary and secondary antibodies were obtained from Proteintech Group, Inc. (Wuhan, China). Proteins bands were detected using an enhanced chemiluminescence substrate (Thermo Fisher Scientific). Band intensities were quantified using ImageJ software. CDKN1A, GADD45A, and BAX signals were normalized to β-actin. Quantification was based on at least three independent biological experiments, and the normalized values were used for statistical analysis.

### 2.12 Statistical analysis

Statistical analyses were performed using GraphPad Prism software (GraphPad Software, USA). Comparisons between two groups were conducted using the unpaired two-tailed Student's t-test. Comparisons among multiple groups were assessed using one-way analysis of variance (ANOVA) followed by Dunnett’s multiple comparisons test, where the treatment groups (BaP, siBRCA1, BaP+siBRCA1) were compared to the Control group. All data are presented as mean ± standard error of the mean (SEM) from at least three independent experiments. A P-value < 0.05 was considered statistically significant. Significance levels are indicated as ***p < 0.001, **p < 0.01, p < 0.05, and ‘ns’ for not significant.

## 3 Results

### 3.1 Network toxicology analysis of benzo[a]pyrene (BaP)

To systematically identify potential molecular links between BaP and oral cancer, we first performed a network toxicology analysis in accordance with the “computational screening–multi-omics anchoring–functional validation” framework (Figure 1). The chemical structure of BaP was retrieved from PubChem and visualized, confirming its polycyclic aromatic hydrocarbon skeleton (Figure 1A), a structural feature consistent with its metabolic activation to DNA-adduct-forming carcinogenicity. ProTox-II annotated BaP as Toxicity Class 4 with an estimated LD_50_ of 316 mg/kg (Figure 1B). The corresponding molecular-weight/dose distributions and endpoint confidence scores relative to class averages are shown in (Figures 1C,D).

**FIGURE 1:**
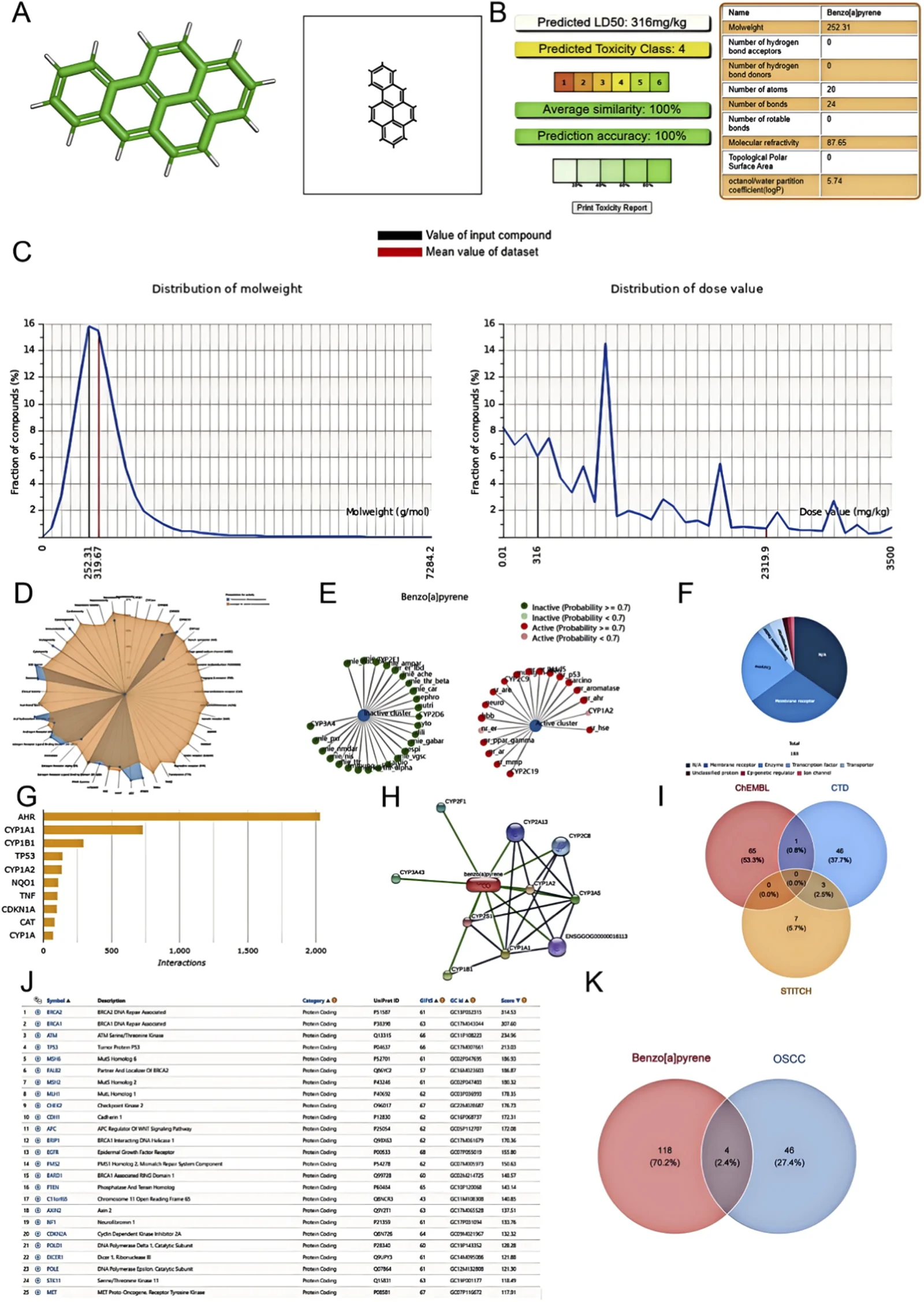
Network toxicology workflow and target identification for benzo[a]pyrene (BaP) **(A)** Three-dimensional chemical structure of BaP retrieved from PubChem. **(B)** Toxicity classification and predicted LD_50_ of BaP based on ProTox-II analysis. **(C)** Distribution of molecular weight and LD_50_ of BaP compared with reference compounds in the database. **(D)** Confidence scores for predicted toxicological endpoints of BaP relative to class averages. **(E)** Predicted biological activities associated with BaP based on network toxicology analysis. **(F–H)** BaP-associated target genes retrieved from the ChEMBL **(F)**, CTD **(G)**, and STITCH **(H)** databases. **(I)** Venn diagram showing the union of BaP-associated target genes from the three databases. **(J)** Oral cancer–associated genes retrieved from the GeneCards database. **(K)** Venn diagram showing the overlap between BaP-associated targets and oral cancer–related genes.

To identify candidate molecular targets, we collected BaP-related targets from ChEMBL (Figure 1F), STITCH (Figure 1H), and SwissTargetPrediction, which were merged after deduplication; BaP–gene associations were concurrently queried from CTD as an additional evidence layer. After integration, a total of 122 unique BaP-related target proteins were retained for downstream analysis (Figure 1I). Oral cancer-associated genes were retrieved from GeneCards, and top-ranked genes by relevance score were selected to constitute a high-confidence disease gene set (Figure 1J). Intersecting the BaP-related targets with oral cancer genes yielded a candidate overlap set (Figure 1K), and these genes were taken forward for subsequent analyses as putative mediators of BaP-associated oral carcinogenesis. Functional annotation of BaP-associated activities highlighted processes consistent with genotoxic stress (DNA damage response), oxidative stress, and cell cycle perturbation (Figure 1E), thus guiding further prioritization at both the gene and pathway levels.

Integration and deduplication of the BaP-associated target sources yielded 122 unique genes. Intersecting this set with the 50 GeneCards genes retained in the original disease-gene analysis yielded exactly four genes—EGFR, HRAS, TP53, and BRCA1 (Figure 1K and Supplementary Table S1). Thus, all four genes represented the complete intersection rather than a subset selected from a larger overlap pool. Updated CTD quantification identified 12, 23, 108, and 32 curated BaP-interaction records for EGFR, HRAS, TP53, and BRCA1, respectively. In the induced four-node STRING network, all four genes had a degree of 3.

Notably, AHR was captured in the upstream BaP-associated target set and showed the largest number of curated BaP–gene interaction records in CTD (Figure 1G). However, AHR was not present in the 50 oral cancer-related genes retained from GeneCards and therefore did not enter the four-gene intersection. Its absence from the downstream intersection resulted from the uniform screening criterion rather than from exclusion based on biological relevance. The CTD values shown in Figure 1G represent curated chemical–gene interaction-record counts and should not be interpreted as protein-network degree or a composite prioritization score. While the GeneCards ranks provide a reproducible quantitative filter, they reflect general oncological research volume and may favor extensively studied cancer genes over less-characterized but potentially mechanistically relevant candidates.

### 3.2 Selection of BRCA1 for focused follow-up and exploratory docking assessment

The intersection analysis identified EGFR, HRAS, TP53, and BRCA1 as four candidates shared between the BaP-associated and oral cancer-related gene sets. The intersection itself did not generate a composite quantitative hierarchy, and CTD interaction counts and STRING degree did not establish BRCA1 as the highest-ranked network candidate. BRCA1 was selected for focused downstream characterization as a study-specific, testable candidate based on the convergence of its HNSC expression pattern (Figure 2A), its pan-cancer expression profile (Figure 2B), and supporting protein-level context (Figures 2C,D), its established role in DNA-damage-response and genome-maintenance pathways, and its suitability for the planned structural and cellular analyses. This decision should not be interpreted as evidence that BRCA1 has greater network centrality or toxicological importance than the other intersection genes.

**FIGURE 2:**
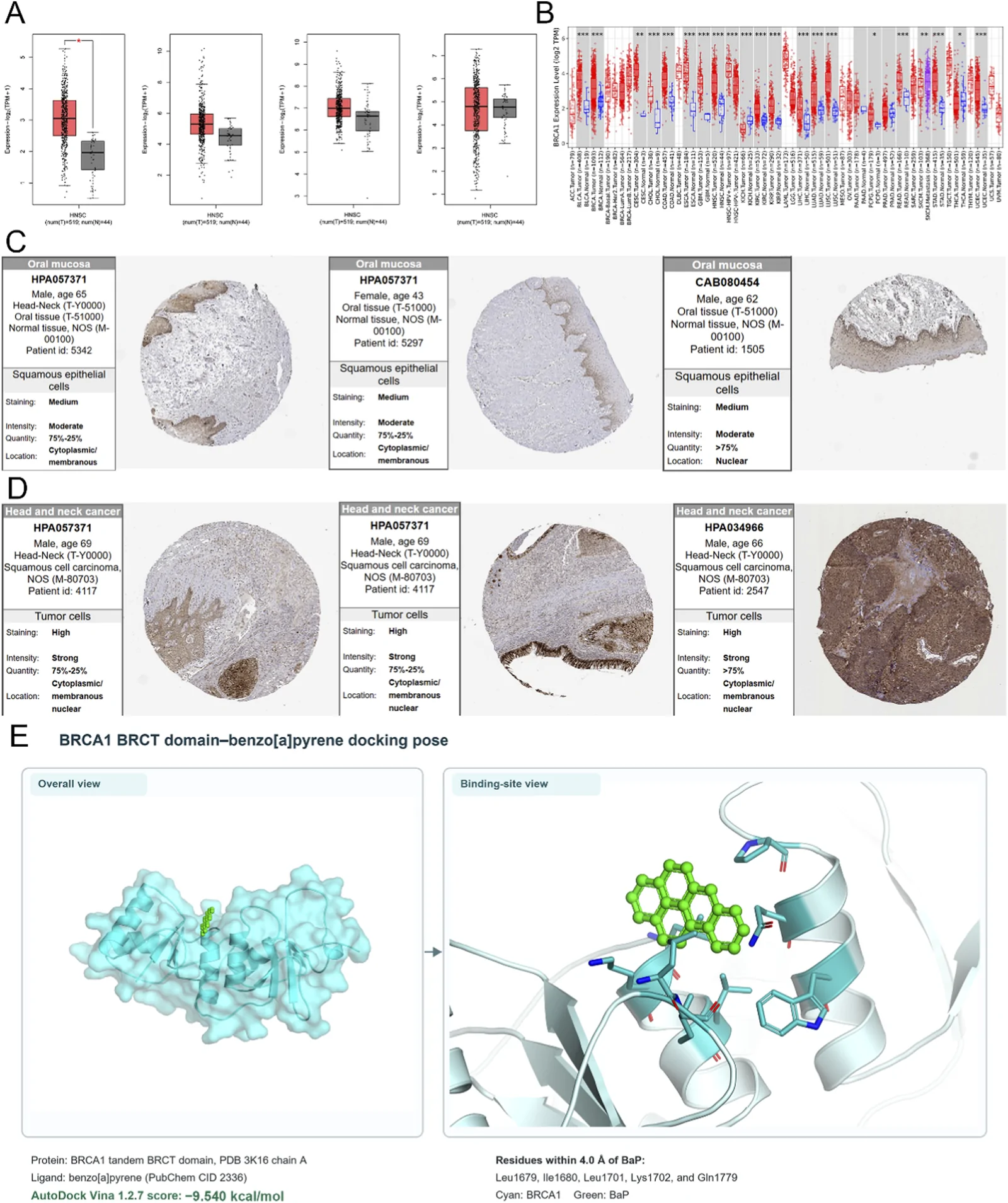
Expression profiling of candidate target genes and docking-based prioritization **(A)** Differential expression of candidate BaP-associated target genes in head and neck squamous cell carcinoma (HNSC) compared with normal tissues (TCGA–GTEx). **(B)** Pan-cancer expression profile of BRCA1 across multiple tumor types. **(C,D)** Immunohistochemical staining of BRCA1 in HNSC tissues **(C)** and normal oral mucosa **(D)** from the Human Protein Atlas. **(E)** Representative molecular docking pose of BaP within the predicted BRCA1 binding site.

Subsequently, we evaluated whether BaP could adopt an energetically favorable binding pose on BRCA1 through molecular docking (Figure 2E). The docking results supported an *in silico* high-affinity binding mode, corroborating BRCA1 as a structurally plausible BaP-associated candidate. Docking indicated that BaP could adopt an energetically favorable pose in the modeled BRCA1 system. This result provided only computational support for structural plausibility and did not demonstrate direct biochemical binding. The docking analysis was therefore used as an exploratory structural assessment rather than as mechanistic or experimental validation.

### 3.3 Expression and distribution of BRCA1 in normal tissues

To understand the biological baseline of BRCA1 beyond tumors, we examined its expression in normal tissues using the Human Protein Atlas (Figure 3). BRCA1 exhibited broad tissue expression, with relatively high levels in lymphoid-associated tissues such as lymph nodes (Figure 3A). Subcellular localization data indicated its nuclear localization (Figure 3B), consistent with the established functions of BRCA1 in genome maintenance. Single-cell summaries further revealed cell-type heterogeneity in BRCA1 expression across different organs (Figure 3C). Moreover, genomic localization of BRCA1 was confirmed on chromosome 17q21 (Figure 3D). These results provide context for interpreting BRCA1 dysregulation in tumors and substantiate the rationale for focusing on BRCA1 in BaP-associated carcinogenesis.

**FIGURE 3:**
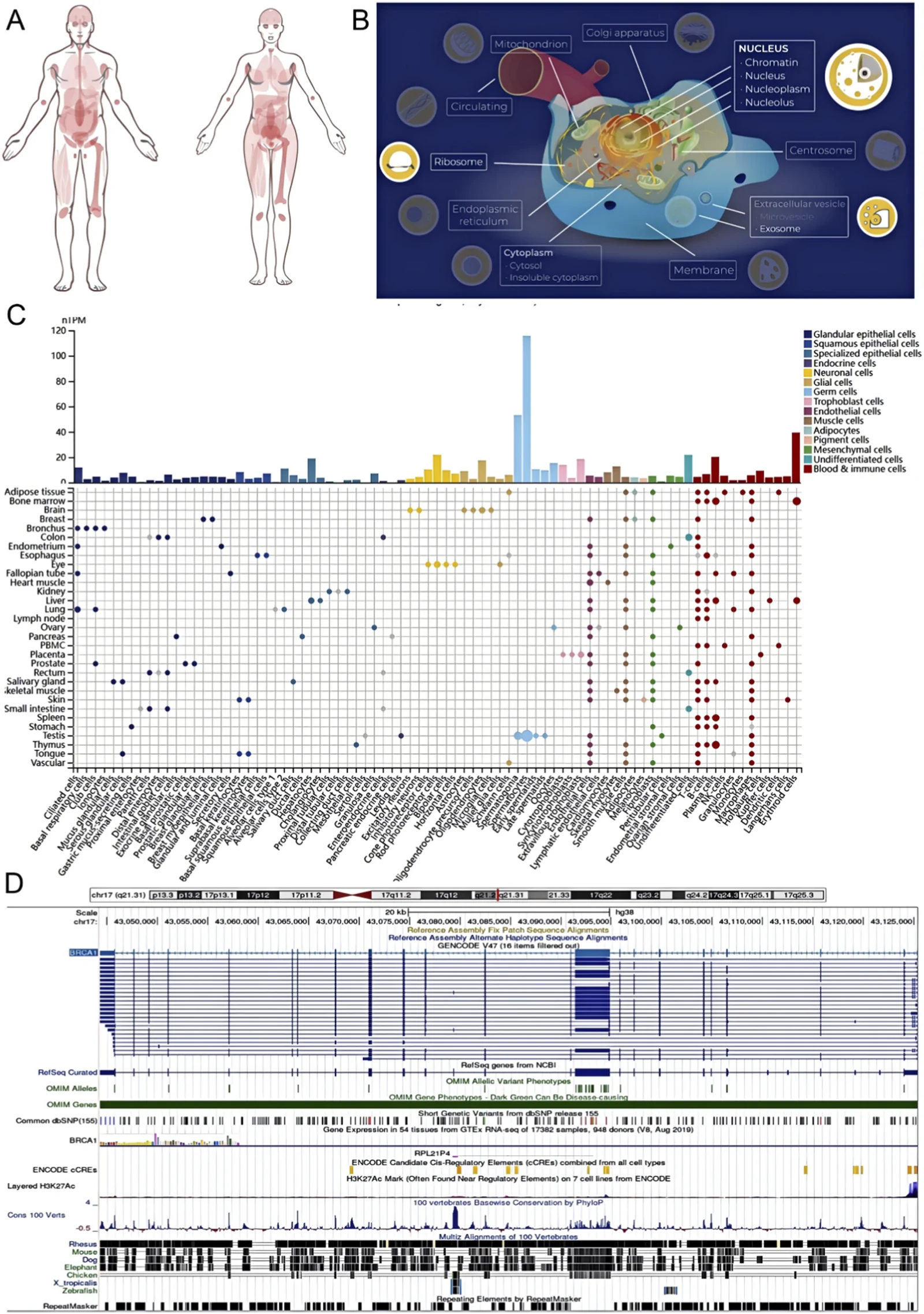
Baseline expression and localization of BRCA1 in normal tissues **(A)** Tissue-level expression distribution of BRCA1 across normal human organs (Human Protein Atlas). **(B)** Subcellular localization of BRCA1 based on curated localization datasets. **(C)** Single-cell expression profiles of BRCA1 across major cell types in normal human tissues. **(D)** Genomic localization of the BRCA1 gene on human chromosome 17q21.

### 3.4 Single-cell and spatial transcriptomic profiling of BRCA1 in oral cancer

To delineate the cellular and spatial architecture of BRCA1 within the oral cancer microenvironment, we evaluated BRCA1 expression at single-cell and spatial resolution using datasets curated by OralDB (Figure 4).

**FIGURE 4:**
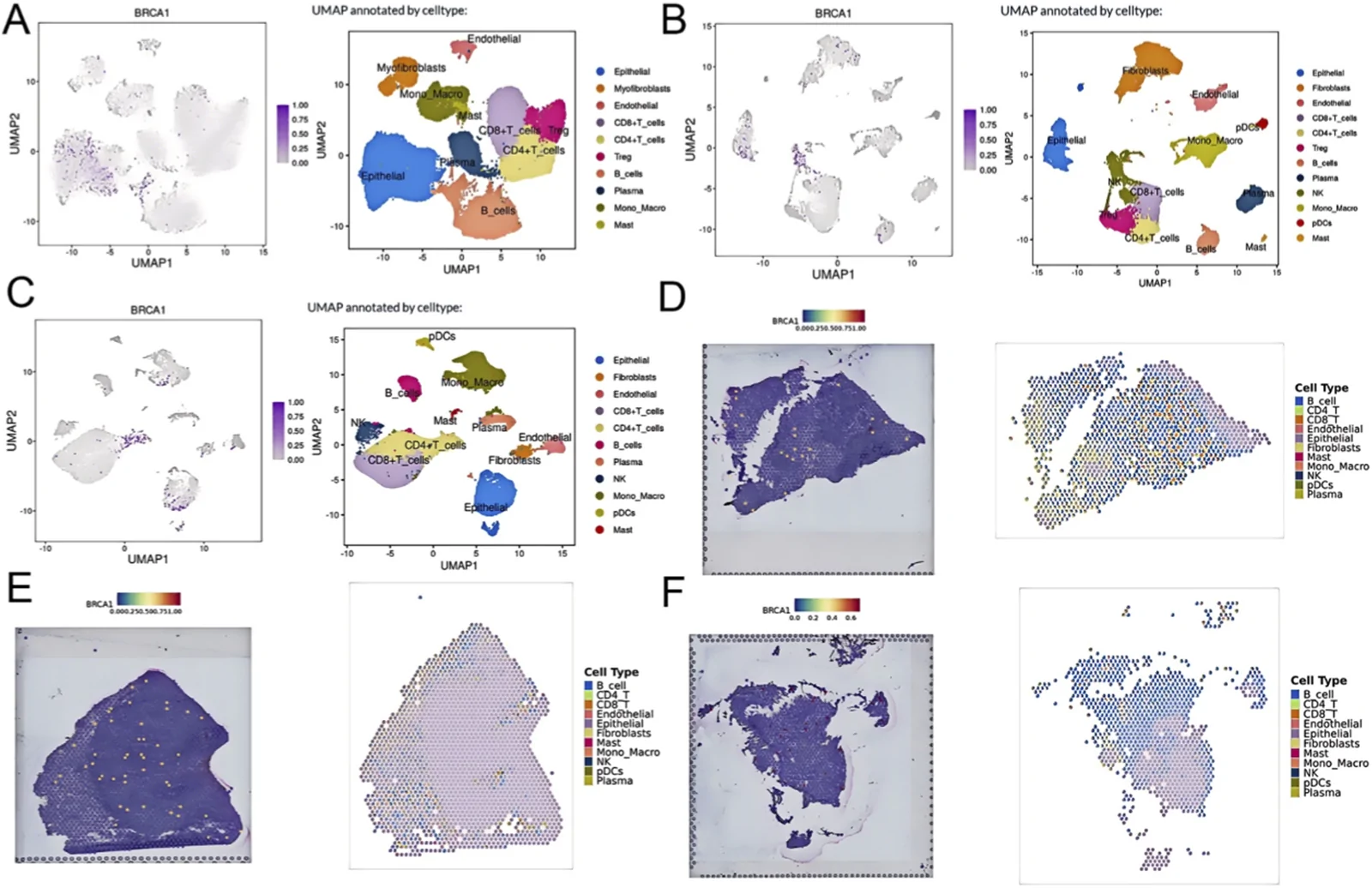
Single-cell and spatial transcriptomic mapping of BRCA1 in oral cancer **(A–C)** Single-cell RNA-sequencing analysis showing BRCA1 expression across cell populations in oral cancer datasets GSE173468 **(A)**, GSE181919 **(B)**, and GSE234933 **(C)**. **(D–F)** Spatial transcriptomic maps of BRCA1 expression in oral cancer tissues from dataset GSE208253, illustrating region-specific expression patterns.

In the scRNA-seq datasets GSE173468, GSE181919, and GSE234933, BRCA1 expression exhibited marked heterogeneity across malignant epithelial cells and immune/stromal compartments (Figures 4A–C). High BRCA1 signals were detectable in subsets of tumor cells and certain immune populations, indicating that BRCA1 expression programs may vary according to cell type within the oral cancer microenvironment. Spatial transcriptomics (GSE208253) further revealed regionalized expression patterns of BRCA1 across tumor sections (Figures 4D–F). BRCA1 signal was detected in specific microanatomical regions overlapping with tumor cell-dense areas and immune-transcript-enriched zones, providing a spatial context for interpreting BRCA1-associated programs in oral cancer. Given that these datasets lack BaP exposure annotation, the single-cell and spatial analyses here primarily serve to characterize the cellular and spatial distribution features of BRCA1 in oral cancer rather than to directly infer BaP-driven microenvironmental dynamics *in vivo*. Given that these datasets do not include BaP exposure metadata, the single-cell and spatial profiling presented here is descriptive in nature and should not be interpreted as evidence for BaP-induced alterations in the tumor microenvironment.

### 3.5 Functional association analyses of BRCA1 in HNSC

To define a more conservative BRCA1 association context, STRING v12.0 was reanalyzed with only experimental and curated-database evidence active (Figure 5A and Supplementary Table S3). The resulting induced network contained 11 nodes and 45 retained edges. BRCA1 was connected to all 10 first-shell partners (degree = 10): BARD1, BRIP1, RBBP8, ABRAXAS1, BABAM1, UIMC1, MRE11, RAD50, TOPBP1, and PALB2. Of the 45 edges, 36 were supported by both experimental and curated-database channels, whereas nine were supported by the curated-database channel alone. Independently, filtering BioGRID v5.0.260 to human multi-validated physical-class records yielded 1,551 BRCA1-anchored records involving 313 non-self partners. Figure 5B displays the 20 partners with the strongest publication-level support, led by BARD1 (86 unique PubMed references), RBBP8 (38), BRIP1 (Jeffy et al., 2000), UBE2D3 (Flores et al., 2025), UIMC1 (Mi et al., 2023), and PALB2 (Li et al., 2025). These evidence-resolved networks place BRCA1 within well-supported DNA-damage-repair and protein-complex contexts; however, the associations do not by themselves establish direct binary physical binding. KEGG/GO enrichment analyses and GSEA based on stratification by BRCA1 expression further pointed to pathways associated with DNA damage repair, cell cycle regulation, and the p53 signaling axis (Figures 5C–F). These results suggest that tumors with high BRCA1 expression are closely linked to transcriptional programs of genome maintenance and checkpoint regulation, providing candidate downstream pathways for experimental validation in the context of BaP.

**FIGURE 5:**
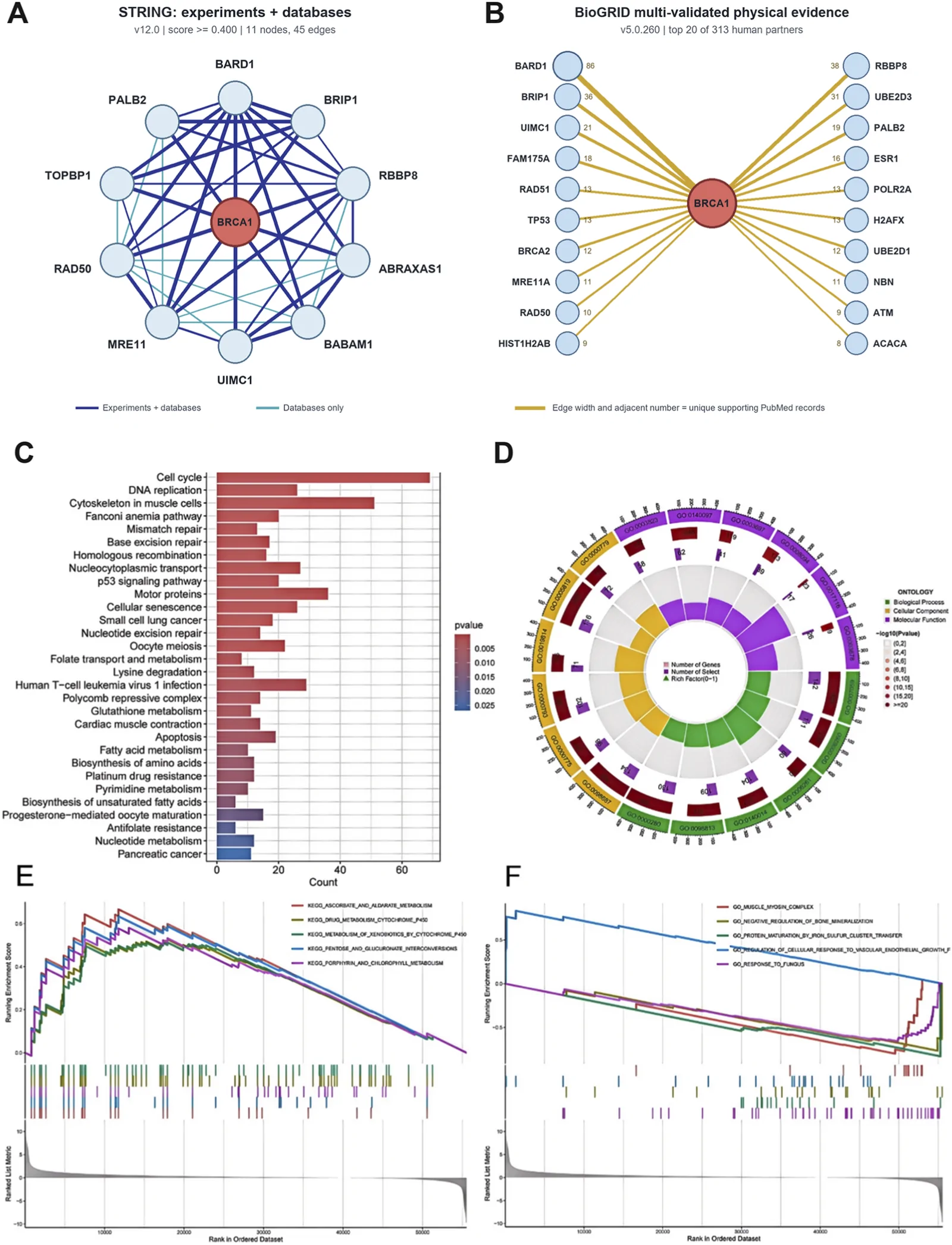
Functional association analysis of BRCA1 in head and neck squamous cell carcinoma **(A)** BRCA1-centered STRING v12.0 protein-association network for *Homo sapiens* generated with a required interaction score of 0.400, 10 first-shell partners, no second-shell partners, and only experiments and curated databases active. The network contains 11 nodes and 45 retained edges; dark-blue edges have support from both active channels and teal edges have curated-database support only. **(B)** BioGRID v5.0.260 network based on human multi-validated physical-class records. The 20 displayed partners were ranked by unique supporting PubMed references; edge width and the adjacent number indicate the number of unique references. Physical-class records may represent direct binding, co-complex recovery, or proximity evidence depending on the experimental system. **(C)** KEGG pathway enrichment analysis of BRCA1-associated genes in HNSC. **(D)** Gene Ontology (GO) enrichment analysis of BRCA1-associated genes in HNSC. **(E,F)** Gene set enrichment analysis (GSEA) of KEGG pathways **(E)** and GO terms **(F)** comparing BRCA1-high and BRCA1-low HNSC samples. Channel-level STRING scores and BioGRID experimental systems and PubMed references are provided in Supplementary Table S3.

### 3.6 Molecular dynamics characterization of the predicted BaP–BRCA1 complex

Building on the structural feasibility obtained from docking, we further evaluated the dynamic stability of the predicted BaP–BRCA1 interaction through molecular dynamics (MD) simulations (Figure 6). These MD observations derive from a single 100-ns trajectory per system and should be interpreted as a descriptive characterization of one simulated structural model rather than as a statistically converged or replicate-validated estimate of binding stability. To provide a broader context for the structural plausibility of BaP binding, we performed standardized docking of BaP against the four candidate proteins (BRCA1, HRAS, EGFR, and TP53) using the same search parameters (Vina scoring function, 24 × 24 × 24 Å box, exhaustiveness = 32, three independent docking runs with different random seeds). The mean top-pose Vina scores (mean ± standard deviation among docking repeats) were: BRCA1 −9.540 ± 0.001 kcal/mol; HRAS −8.986 ± 0.008; EGFR −6.928 ± 0.012; and TP53–5.222 ± 0.011(Table Supplementary S2). These scores indicate that, *in silico*, BaP can adopt energetically favorable poses for all four targets, with BRCA1 showing the most favorable score in this comparative exercise. However, docking scores do not predict transcriptional effects and should not be interpreted as evidence of selective binding in cells. The backbone RMSD of the predicted BRCA1–BaP complex averaged 0.160 ± 0.025 nm across the complete 100 ns trajectory. During the final 20 ns, the RMSD fluctuated around 0.180 ± 0.018 nm. The 95th percentile in this interval was 0.212 nm, and all values remained below 0.250 nm. These results indicate a relatively bounded fluctuation regime during the final stage of the simulation, although complete trajectory-wide convergence cannot be inferred (Figure 6A). The principal contact residues identified across representative snapshots were Asp1778, Gln1779, Trp1782, and Gln1785. Their RMSF values were 0.1007, 0.0871, 0.0869, and 0.0889 nm, respectively, corresponding to a range of 0.0869–0.1007 nm(Figure 6B). Hydrogen bond analysis supported the presence of persistent intermolecular contacts during the simulation (Figure 6C). Rg analysis revealed subtle conformational adjustments upon ligand binding (Figure 6D), and free energy landscape analysis indicated stable conformational states of the complex (Figures 6E,F). Representative snapshots showed BaP persistently occupying the predicted binding pocket (Figures 6G,H). The mean short-range Coulombic interaction energy between BRCA1 and BaP was −7.21 ± 6.60 kJ/mol, while the Lennard–Jones interaction energy was −73.70 ± 14.94 kJ/mol. Their instantaneous sum averaged −80.91 ± 16.87 kJ/mol (Figures 6I,J). These values, extracted from the GROMACS energy module, describe nonbonded contacts within the simulated trajectory; they do not include polar solvation, nonpolar solvation, or entropic terms, and therefore should not be interpreted as MM-PBSA or thermodynamic binding free energies. The reported uncertainties (Err.Est.) are block-averaging estimates of the uncertainty in the trajectory mean, not standard deviations across independent replicas. Collectively, these analyses characterized the dynamic behavior of the predicted BaP–BRCA1 model and provided computational evidence of structural plausibility. They do not demonstrate direct physical binding and should not be interpreted as an explanation for the observed increase in BRCA1 mRNA.

**FIGURE 6:**
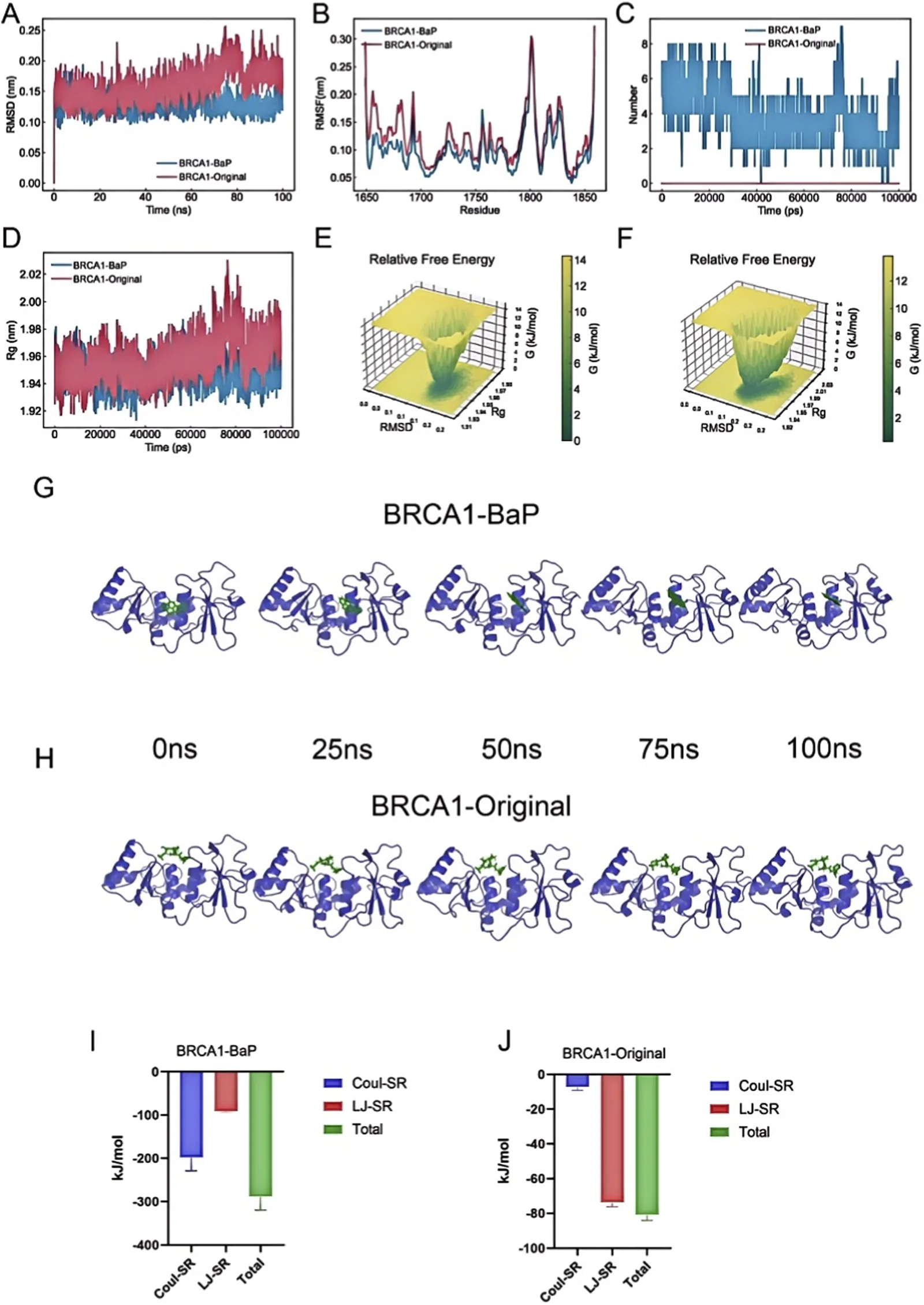
Molecular dynamics characterization of the predicted BaP–BRCA1 complex **(A)** Root mean square deviation (RMSD) trajectories of BRCA1–co-crystallized phosphopeptide and BRCA1–BaP complex over 100 ns. **(B)** Root mean square fluctuation (RMSF) per residue for BRCA1–phosphopeptide complex and BaP-bound BRCA1. **(C)** Number of intermolecular hydrogen bonds during simulation. **(D)** Radius of gyration (Rg) of BRCA1 in the phosphopeptide-bound and BaP-bound states. **(E,F)** Free energy landscapes (FEL) of the BRCA1–BaP complex **(E)** and BRCA1–phosphopeptide complex **(F)**. **(G,H)** Representative structural snapshots of the BRCA1–BaP complex **(G)** and BRCA1–phosphopeptide complex **(H)** at selected simulation time points. **(I,J)** Short-range nonbonded interaction-energy components (Coul-SR and LJ-SR) for the simulated BRCA1–BaP system. Error bars represent block-averaging uncertainty estimates (Err.Est.) from gmx energy, calculated over five 20-ns blocks; they are not standard deviations among independent simulation replicates. These components are descriptive interaction energies and are not MM-PBSA or thermodynamic binding free energies. All analyses shown in this figure are computational; they describe the behavior of a single predicted structural model and do not constitute biochemical evidence of direct BaP–BRCA1 binding.

To evaluate the reproducibility of the trajectory-level observations, two additional independent 100-ns MD simulations were performed using different initial velocity seeds, providing three independent trajectories and a cumulative production sampling time of 300 ns (Supplementary Table S1). For each scalar metric, a time-averaged value was first calculated separately for each trajectory, and the mean and sample standard deviation were subsequently calculated across the three independent runs.

The run-averaged backbone RMSD values were 0.127, 0.156, and 0.151 nm for Runs 1–3, respectively, yielding an across-run mean of 0.145 ± 0.015 nm (n = 3; Supplementary Figure S1A). Although Runs 2 and 3 exhibited moderately higher RMSD values during portions of the latter half of the simulations, all three trajectories remained within a broadly comparable fluctuation range. The corresponding mean Rg values were 1.948, 1.951, and 1.944 nm, resulting in an across-run mean of 1.948 ± 0.004 nm (n = 3; Supplementary Figure S1B).

The run-averaged hydrogen-bond counts were 4.068, 3.809, and 3.422 for the three trajectories, respectively, giving an across-run mean of 3.766 ± 0.325 hydrogen bonds (n = 3; Supplementary Figure S1C). Hydrogen bonds were dynamically formed and disrupted throughout the simulations, with observed counts ranging from 0 to 9, 0 to 8, and 0 to 8 in Runs 1–3, respectively. During the final 20 ns, the corresponding mean hydrogen-bond counts were 2.959, 3.711, and 3.490, yielding an across-run mean of 3.387 ± 0.387.

The residue-averaged RMSF values were 0.100, 0.152, and 0.138 nm, with an across-run mean of 0.130 ± 0.027 nm. The pairwise correlations between the three residue-level RMSF profiles ranged from 0.931 to 0.979, indicating that the principal locations of protein flexibility were reproducible, although the magnitude of the fluctuations varied among simulations (Supplementary Figure S1D). The highest mean fluctuations occurred around residues 1801–1802, whereas the largest between-run RMSF variability was observed at residue 1796.

Collectively, the replicate analyses reproduced the principal qualitative conformational patterns while demonstrating measurable run-to-run variability. The independent simulations reduce dependence on a single initial velocity assignment but do not establish complete conformational or thermodynamic convergence or provide evidence of direct biochemical binding between BaP and BRCA1.

### 3.7 BaP-associated proliferation is attenuated by BRCA1 knockdown and accompanied by changes in p53-axis readouts

To functionally test the hypotheses generated by computational analyses, we examined the effects of BaP in CAL27 oral cancer cells (Figure 7). CCK-8 assays demonstrated that BaP exposure increased cell viability in a concentration- and time-dependent manner (Figures 7A–C). To assess dependence on BRCA1, BRCA1 was silenced by siRNA, which reduced proliferative capacity. Notably, BaP co-treatment failed to restore proliferation to control levels, suggesting that BRCA1 is required for the full BaP-driven growth phenotype. qRT-PCR further showed that BaP exposure induced upregulation of BRCA1 mRNA (Figure 7D). Concomitantly, classic p53 downstream targets (CDKN1A, GADD45A, and BAX) were downregulated following BaP treatment (Figures 7E–G). BaP treatment was accompanied by increased BRCA1 mRNA expression, whereas BRCA1 knockdown attenuated the BaP-associated proliferative response. These observations support the functional involvement of BRCA1 under the tested conditions. However, they do not establish whether the increase in BRCA1 expression is directly induced by BaP or mediated indirectly through a broader DNA-damage or cellular stress response, and they do not establish a corresponding causal relationship in patients.

**FIGURE 7:**
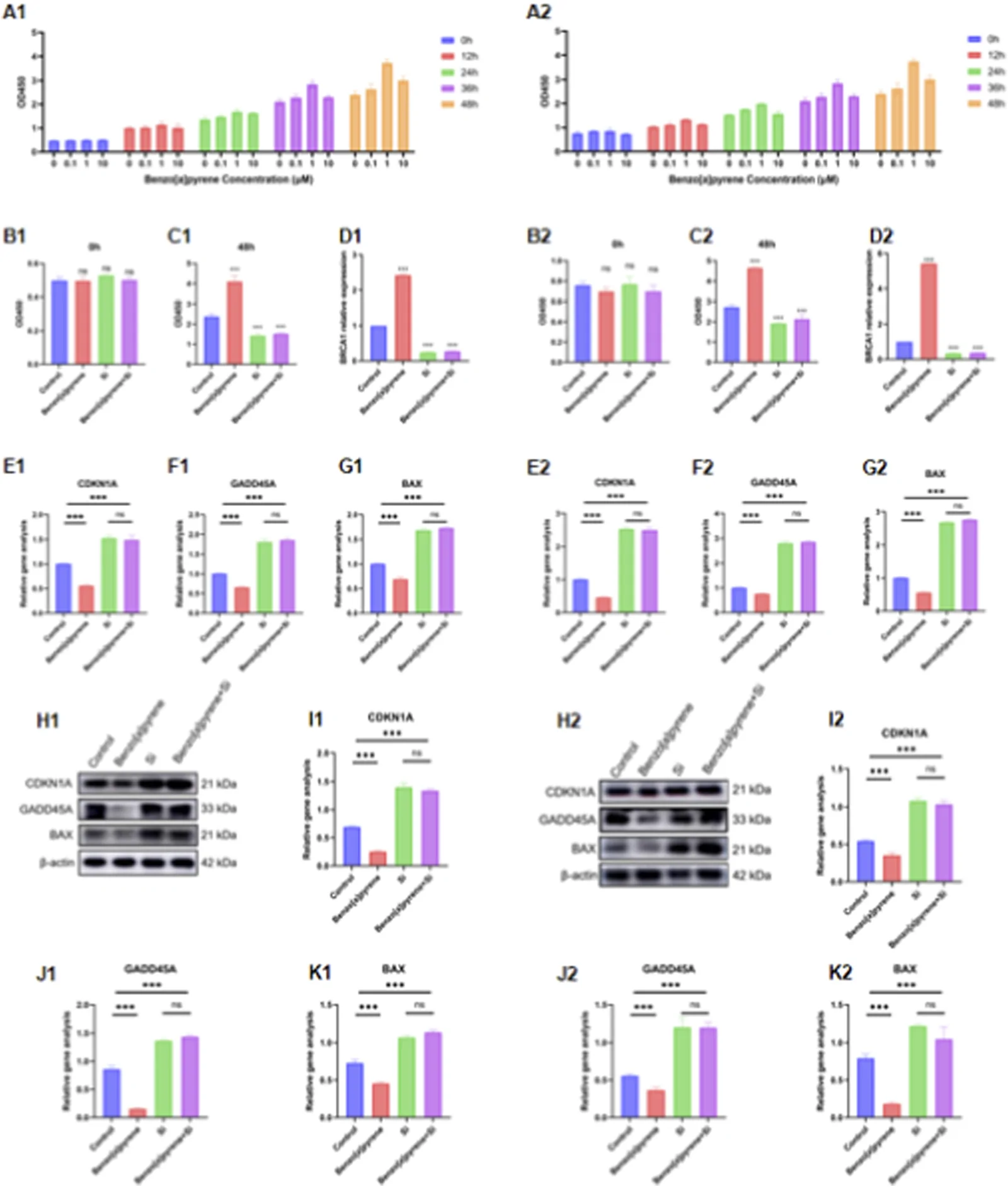
BaP-associated proliferation and p53-axis transcript changes in OSCC cells are modulated by BRCA1 expression **(A1–A2)** CCK-8 assays (OD450) for CAL27 **(A1)** and SCC9 **(A2)** cells treated with BaP (0, 0.1, 1, 10 μM) for the indicated time points (0–48 h). **(B1–B2)** Baseline OD450 measurements at 0 h in CAL27 **(B1)** and SCC9 **(B2)** under the indicated conditions. **(C1–C2)** OD450 at 48 h in CAL27 **(C1)** and SCC9 **(C2)** under control, BaP (1 μM), BRCA1 siRNA (siBRCA1), and BaP + siBRCA1 conditions. **(D1–D2)** qRT-PCR analysis of BRCA1 mRNA levels in CAL27 **(D1)** and SCC9 **(D2)** following BaP exposure and/or BRCA1 knockdown. **(E1–G2)** qRT-PCR analysis of p53-axis downstream genes CDKN1A **(E1–E2)**, GADD45A **(F1–F2)**, and BAX **(G1–G2)** in CAL27 (left panels) and SCC9 (right panels) under the indicated treatments. **(H1–H2)** Representative Western blots of CDKN1A, GADD45A, and BAX in CAL27 **(H1)** and SCC9 **(H2)** cells; β-actin served as a loading control. **(I1–K2)** Densitometric quantification of Western blot signals for CDKN1A **(I1–I2)**, GADD45A **(J1–J2)**, and BAX **(K1–K2)**, normalized to β-actin. Data are presented as mean ± SEM. Statistical significance is indicated as ***p < 0.001; ns, not significant. Statistical analyses were performed using GraphPad Prism. Comparisons among multiple groups were assessed using one-way ANOVA followed by Dunnett’s multiple comparisons test, and comparisons between two groups were evaluated using the unpaired two-tailed Student's t-test.

## 4 Discussion

This hypothesis-generating and methodologically oriented exploratory study developed an integrated framework combining computational target prioritization, multi-omics contextualization, and preliminary phenotypic assessment. The principal contribution is the screening strategy itself rather than definitive validation of BRCA1 as a mechanistic mediator of BaP-associated oral carcinogenesis. The predefined intersection yielded EGFR, HRAS, TP53, and BRCA1 without imposing a composite hierarchy, and BRCA1 was selected as a study-specific, testable candidate for focused follow-up. The computational, transcriptomic, and cellular analyses address different questions and differ in evidential scope; they should not be interpreted as mutually confirmatory proof of a single causal mechanism (Wei et al., 2023; Mi et al., 2023).

The initial intersection analysis did not generate a composite quantitative hierarchy among EGFR, HRAS, TP53, and BRCA1. The four genes constituted the complete overlap defined by the prespecified target- and disease-gene sets, and all showed identical degree values in the induced four-node STRING network. BRCA1 was selected as an experimentally tractable working candidate for the present hypothesis-generating study. Its selection does not exclude alternative mediators and should not be interpreted as demonstrating superior network centrality or toxicological importance.

AHR is a canonical mediator of BaP sensing and metabolic activation and therefore provides essential biological context for interpreting BaP toxicity (Bukowska et al., 2022; Pandelides et al., 2023; Park et al., 2017). The present screening framework, however, was designed to identify complementary candidates by intersecting independently derived BaP-associated and oral cancer-related gene sets rather than to reselect established mediators according to prior mechanistic knowledge. Within this framework, AHR remained strongly supported as a BaP-associated gene but did not meet the disease-set intersection criterion. BRCA1 should therefore be regarded as a complementary, data-derived candidate selected for focused hypothesis generation rather than as a replacement for AHR or as evidence of greater mechanistic importance.

### 4.1 Distinguishing structural plausibility from the functional involvement of BRCA1

A central point requiring clarification is that the computational and experimental analyses address distinct questions. Molecular docking and molecular dynamics simulations were used to assess whether BaP could adopt a structurally plausible configuration in the modeled BRCA1 system. These calculations do not demonstrate direct binding and do not predict the direction of BRCA1 transcriptional regulation (Zainulabidin et al., 2025; Zia et al., 2024).

In contrast, the cell experiments showed that BaP treatment was accompanied by increased BRCA1 mRNA expression and that BRCA1 knockdown attenuated the associated proliferative response. These observations support the functional involvement of BRCA1 in the cellular response to BaP under the tested conditions. However, they do not establish that a direct physical interaction between BaP and BRCA1 causes BRCA1 upregulation or cell proliferation.

An indirect stress-response mechanism may provide a plausible explanation for the transcriptional observation. BaP metabolites can generate DNA adducts, oxidative stress, and replication stress (Yan et al., 2011; Zou et al., 2024). These perturbations may activate upstream DNA-damage-response regulators, including ATM and ATR, which could alter BRCA1 expression or activity (Chang et al., 2021; Bermúdez-Guzmán, 2021). The observed increase in BRCA1 mRNA may therefore represent an adaptive response to BaP-associated genotoxic stress. Because ATM/ATR signaling and other candidate upstream pathways were not directly tested here, these explanations remain hypotheses.

Accordingly, structural plausibility and functional involvement are regarded as two independent, hypothesis-generating observations. BRCA1 is presented as a candidate functional node in the cellular response to BaP rather than as an established direct receptor or molecular target of BaP.

### 4.2 BaP exposure is associated with reduced p53-axis downstream readouts and potential implications

An unexpected and noteworthy finding of this study was that BaP treatment, despite promoting cell proliferation, was accompanied by transcriptional downregulation of classical p53 downstream target genes (CDKN1A, GADD45A, BAX) (Labib et al., 2012). This stands in stark contrast to the canonical acute DNA damage response, which typically activates p53 and upregulates pro-apoptotic/cycle-arrest genes (Hafner et al., 2019). However, because p53 abundance, activation state, and post-translational modifications were not measured, these results do not demonstrate direct suppression of p53 signaling or direct inhibitory regulation by BRCA1. They instead identify an association among BaP treatment, BRCA1 status, and p53-axis downstream readouts that requires mechanistic validation. The altered downstream readouts may reflect a pro-survival state under chronic or sub-toxic BaP exposure, but the potential involvement of ATM/ATR, MDM2, NF-κB, AP-1, or other stress-response pathways remains hypothetical (Jiang et al., 2011; Flores et al., 2025; Shi et al., 2021). Knockdown of BRCA1 was accompanied by partial restoration of these readouts; this finding does not establish that BRCA1 directly inhibits the p53 pathway, but rather suggests that BRCA1 status may be associated with changes in p53-axis downstream outputs through mechanisms yet to be defined (Nelson et al., 2023). These observations warrant further investigation, including direct measurement of p53 protein stability, abundance, activation state, and post-translational modifications.

### 4.3 Methodological contributions and limitations

Methodologically, this workflow demonstrates a strategy to systematically narrow the hypothesis space in environmental carcinogen research. By integrating multiple public target databases, tissue-level and single-cell resolution expression filtering, we reduced false-positive rates in computational predictions; the inclusion of spatial transcriptomics further revealed intra-tumoral regional heterogeneity of BRCA1 expression, a dimension rarely addressed in conventional toxicology studies (Berrell et al., 2024; Lee et al., 2024; Jeffy et al., 2000).

However, this study has clear limitations, which also define priorities for future research. First, causality has not been established. Owing to the lack of BaP exposure metadata in public single-cell datasets, our multi-omics analyses could only delineate the expression context of BRCA1 in oral cancer, without inferring toxicant-driven dynamics *in vivo*. Second, direct interaction evidence is lacking. As noted above, computational simulations cannot substitute for biochemical validation. The short-range Coulombic and Lennard–Jones energy terms reported here provide descriptive information about nonbonded contacts within the simulated systems but do not estimate thermodynamic binding free energy. Third, the experimental validation was limited in depth. Our *in vitro* experiments were confined to two cell lines (CAL27 and SCC9), employed transient partial knockdown strategies, and did not assess the modification status of BRCA1 or p53 at the protein level, restricting precise mechanistic resolution. Fourth, the GeneCards relevance score is influenced by general publication volume for gene–disease associations rather than by oral cancer- or BaP-specific mechanistic evidence. This may introduce a bias toward well-studied oncogenes (e.g., TP53, BRCA1, EGFR) and could potentially overshadow lesser-studied genes that might be more specifically relevant to BaP-driven carcinogenesis. Readers should therefore interpret the GeneCards-based filtering as a reproducible bibliometric criterion rather than as evidence of exposure-specific pathogenic importance. Fifth, STRING-based connectivity metrics are susceptible to inflation from text-mining and co-occurrence channels, particularly for well-studied genes. We have now restricted the evidence sources to experiments and databases; nevertheless, the resulting conservative network still exhibited limited discriminative power among the four intersection genes, underscoring that network degree alone is insufficient to prioritize candidates in this specific gene set. Sixth, following the discovery of the label-swap and terminology errors in Figure 6, we conducted a systematic re-audit of all other quantitative outputs derived from the same computational pipelines, including docking scores (Figure 2E and Supplementary Table S2), STRING/BioGRID network data (Figures 5A,B), and GSEA rankings (Figures 5E,F). All values were re-verified against their original source files and computational logs, confirming that the Figure 6 errors were isolated incidents related to figure assembly and terminology rather than symptoms of a broader data-handling gap.

Mendelian randomization is an effective tool for establishing causal relationships (Peng et al., 2025). We also considered whether Mendelian randomization could strengthen causal inference. However, we were unable to identify validated genetic instruments or GWAS summary statistics that specifically represent BaP exposure or internal dose. Smoking-related variants are not specific to BaP, while xenobiotic-metabolism variants may affect cancer phenotypes through multiple pathways. These indirect proxies may therefore violate the instrumental-variable assumptions required for a valid MR analysis. For this reason, MR was not performed, and the BaP–BRCA1 relationship remains a hypothesis requiring direct experimental and exposure-annotated clinical validation.

The molecular dynamics simulations were based on a single 100-ns trajectory per system, without independent replicas. Thus, the observed conformational behavior, although stable within this particular trajectory, cannot be regarded as a statistically robust estimate of binding stability or as evidence of thermodynamic convergence. Time-block analysis provided descriptive consistency checks but does not substitute for replicate simulations or direct biophysical assays. Therefore, all MD-derived observations remain hypothesis-generating and require validation by independent computational replicas and by biochemical methods such as surface plasmon resonance or isothermal titration calorimetry.

Accordingly, all mechanistic interpretations proposed in this study should be regarded as provisional and hypothesis-generating. The computational observations require direct biochemical validation, the cellular findings require confirmation in additional experimental models, and the clinical relevance requires assessment in exposure-annotated human cohorts.

### 4.4 Future perspectives

To translate our hypothesis into a definitive mechanistic model, subsequent studies must employ surface plasmon resonance (SPR) or cellular thermal shift assay (CETSA) to verify whether BaP directly binds to BRCA1; use conditional knockout mouse models or patient-derived xenografts (PDX) to test *in vivo* the dependency of BaP carcinogenesis on BRCA1; and apply phosphoproteomics to map the activation landscape of upstream DDR kinases (ATM/ATR) in response to BaP. Furthermore, exploring whether oral cancer patients with high BRCA1 expression are more sensitive to PARP inhibitors would add translational value to our findings (Gedeonová et al., 2025). Future clinical studies should prioritize exposure-annotated cohorts with paired measurements of BaP metabolites or BPDE-DNA adducts and BRCA1 expression. Such data would permit a more direct assessment of whether BaP-associated internal exposure is associated with BRCA1 alterations in oral cancer tissues.

## 5 Conclusion

In conclusion, this hypothesis-generating exploratory study presents an integrated framework that identified EGFR, HRAS, TP53, and BRCA1 as the complete intersection of the predefined BaP-associated and oral cancer-related gene sets and selected BRCA1 as a testable candidate for focused follow-up. Exploratory computational analyses provided structural context for a predicted BaP–BRCA1 model, whereas transcriptomic analyses described the expression context of BRCA1 and cell experiments independently showed that BaP treatment was accompanied by increased BRCA1 mRNA expression and that BRCA1 knockdown attenuated BaP-associated proliferation. These evidence streams are mechanistically distinct and do not establish direct BaP–BRCA1 binding, direct regulation of BRCA1 by BaP, a definitive *in vivo* mechanism, or clinical causality. Further biochemical studies, additional experimental models, and exposure-annotated human cohorts are required to test these separate hypotheses. The principal contribution of this work is therefore a transferable strategy for candidate prioritization and hypothesis generation in environmental carcinogen research.

## Data Availability

The original contributions presented in the study are included in the article/Supplementary Material, further inquiries can be directed to the corresponding author.
