# Supplementary material for: An integrated network toxicology and multi-omics framework prioritizes BRCA1 as a testable candidate in benzo[a]pyrene-associated oral cancer
Source: Front Pharmacol. 2026 Sep 15;17:1940046. doi: 10.3389/fphar.2026.1940046 (PMC13619942; doi:10.3389/fphar.2026.1940046)
Supplement: Supplementary file 1 [file DataSheet1.docx]

**Figure S1. Independent molecular dynamics replicate analyses of the predicted BRCA1–BaP complex.**

Three independent 100-ns production simulations were performed using identical simulation parameters but different initial velocity seeds. (A) Backbone root mean square deviation (RMSD) during the three trajectories. (B) Radius of gyration (Rg). (C) Number of intermolecular hydrogen bonds between BRCA1 and BaP. (D) Per-residue root mean square fluctuation (RMSF). The replicate trajectories were analyzed using identical atom selections, fitting procedures, and sampling intervals. The simulations showed broadly reproducible structural fluctuation patterns together with measurable run-to-run variability. These analyses describe the behavior of the predicted complex in the simulated systems and do not constitute evidence of direct biochemical binding or thermodynamic convergence.

**Table S1. Quantitative evidence for all genes in the BaP–oral cancer overlap set**

| Gene | CTD BaP interaction records, n¹ | CTD rank among BaP-associated genes¹ | Unique CTD PubMed references, n¹ | Archived GeneCards rank² | Archived GeneCards relevance score² | STRING degree³ | Original screening outcome |
| --- | --- | --- | --- | --- | --- | --- | --- |
| EGFR | 12 | 99 | 8 | 13 | 155.80 | 1 | Included in the complete four-gene intersection |
| HRAS | 23 | 40 | 12 | 26–50 | <117.91⁴ | 1 | Included in the complete four-gene intersection |
| TP53 | 108 | 4 | 67 | 4 | 213.03 | 1 | Included in the complete four-gene intersection |
| BRCA1 | 32 | 22 | 13 | 2 | 307.60 | 1 | Included in the complete four-gene intersection; selected for focused follow-up |
|  |  |  |  |  |  |  |  |

¹ CTD does not provide a gene-specific 'relevance score' for a chemical–gene relationship. Therefore, the directly reproducible CTD metrics reported here are the number of curated Benzo(a)pyrene–gene interaction records, the rank based on that count, and the number of unique supporting PubMed references. These values were recalculated from the official CTD chemical–gene interaction bulk report generated on 30 July 2026. Counts include all organisms represented in CTD, consistent with the descriptive interaction-count analysis shown in Figure 1G.

² The GeneCards ranks and scores were recovered from the archived GeneCards results shown in Figure 1J of the original manuscript. BRCA1, TP53, and EGFR were visible in the archived top-25 panel. The original full GeneCards export and its database-version metadata were not retained.

³ STRING degree was recalculated in the induced network containing EGFR, HRAS, TP53, and BRCA1 using STRING v12.0 for Homo sapiens, a required interaction score of 0.400, and only the experiments and curated-databases channels active (accessed 21 August 2026). Only BRCA1-TP53 and EGFR-HRAS were retained, so every node had a degree of 1. By comparison, the original all-channel analysis returned all six possible pairwise associations and a degree of 3 for every node. The conservative network therefore changed topology but remained non-discriminative and was not used to prioritize BRCA1.

⁴ HRAS was present in the complete four-gene intersection, confirming that it was within the retained GeneCards top 50. However, the archived Figure 1J displayed only ranks 1–25. Consequently, the exact historical HRAS score cannot be recovered from the retained material. Because the 25th-ranked gene had a score of 117.91, the only defensible historical value is <117.91. A later GeneCards score was not substituted because database scores are version- and query-dependent.

Original inclusion rule: the union of 122 BaP-associated targets was intersected with the 50 GeneCards genes retained in the original analysis. The intersection contained exactly EGFR, HRAS, TP53, and BRCA1. No CTD-count cutoff, STRING-degree cutoff, or retrospective composite score was used to reduce a larger overlap set to these four genes.

**Table S2. Molecular docking results of BaP with target proteins**

| Target | PDB | Vina score, mean ± SD (kcal/mol) | Best score |
| --- | --- | --- | --- |
| BRCA1 | 3K16 | −9.540 ± 0.001 | −9.541 |
| HRAS | 4URZ | −8.986 ± 0.008 | −8.993 |
| EGFR | 1M17 | −6.928 ± 0.012 | −6.942 |
| TP53 | 2XWR | −5.222 ± 0.011 | −5.230 |

**Table S3.** **Evidence-resolved STRING and BioGRID analysis of BRCA1**

This workbook preserves the raw channel-level scores and BioGRID evidence records used to reconstruct Figure 5A-B. STRING associations are not automatically equivalent to direct binary physical binding.

**Table S3A. Analysis parameters and data sources**

| Item | Value | Item | Value | Source | URL |
| --- | --- | --- | --- | --- | --- |
| Analysis date | 2026-08-21 | STRING version | 12.0 | STRING score documentation | https://string-db.org/help/scores/ |
| STRING species | Homo sapiens (NCBI taxon 9606) | Required score | 0.4 | STRING API | https://string-db.org/api/tsv/interaction_partners?identifiers=BRCA1&species=9606&limit=2000&required_score=0&network_type=functional&caller_identity=codex_brca1_revision |
| Active channels | experiments; curated databases | Inactive channels | text mining; co-expression; phylogenetic co-occurrence; gene neighborhood; gene fusion | BioGRID dataset | https://downloads.thebiogrid.org/File/BioGRID/Latest-Release/BIOGRID-MV-Physical-LATEST.tab3.zip |
| STRING Figure 5A | 11 nodes; 45 retained edges | Network type | functional association | BioGRID release | 5.0.260 |
| BioGRID Figure 5B | Top 20 of 313 partners | Physical evidence records | 1551 | BioGRID compilation date | 2026-07-25 |
| BioGRID filter | BRCA1-anchored; physical-class; self-interactions excluded | Figure selection | Top 20 partners ranked by unique PubMed count, then experimental-system count, record count, and gene symbol | BioGRID format | Tab 3.0 multi-validated physical |
| Score prior | 0.041 | Score threshold | 0.4 | Formula | STRING probabilistic channel integration |

**Table S3B. STRING four-gene induced network: channel-resolved edge scores**

| Gene A | Gene B | Experimental score | Database score | Restricted combined score | Retained >=0.400 | Evidence class | All-channel score | Source URL |
| --- | --- | --- | --- | --- | --- | --- | --- | --- |
| EGFR | BRCA1 | 0.103 | 0.0 | 0.103 | No | Experiments only | 0.8 | https://string-db.org/api/tsv/network?identifiers=EGFR%0DHRAS%0DTP53%0DBRCA1&species=9606&required_score=0&network_type=functional&caller_identity=codex_brca1_revision |
| EGFR | HRAS | 0.315 | 0.9 | 0.9285714285714286 | Yes | Experiments + databases | 0.994 | https://string-db.org/api/tsv/network?identifiers=EGFR%0DHRAS%0DTP53%0DBRCA1&species=9606&required_score=0&network_type=functional&caller_identity=codex_brca1_revision |
| HRAS | BRCA1 | 0.0 | 0.0 | 0.0 | No | No active-channel support | 0.528 | https://string-db.org/api/tsv/network?identifiers=EGFR%0DHRAS%0DTP53%0DBRCA1&species=9606&required_score=0&network_type=functional&caller_identity=codex_brca1_revision |
| TP53 | BRCA1 | 0.895 | 0.5 | 0.9452554744525548 | Yes | Experiments + databases | 0.999 | https://string-db.org/api/tsv/network?identifiers=EGFR%0DHRAS%0DTP53%0DBRCA1&species=9606&required_score=0&network_type=functional&caller_identity=codex_brca1_revision |
| TP53 | EGFR | 0.329 | 0.0 | 0.329 | No | Experiments only | 0.943 | https://string-db.org/api/tsv/network?identifiers=EGFR%0DHRAS%0DTP53%0DBRCA1&species=9606&required_score=0&network_type=functional&caller_identity=codex_brca1_revision |
| TP53 | HRAS | 0.292 | 0.0 | 0.292 | No | Experiments only | 0.925 | https://string-db.org/api/tsv/network?identifiers=EGFR%0DHRAS%0DTP53%0DBRCA1&species=9606&required_score=0&network_type=functional&caller_identity=codex_brca1_revision |

**Table S3C. Figure 5A STRING BRCA1-centered conservative induced network**

| Gene A | Gene B | Experimental score | Database score | Restricted combined score | Retained >=0.400 | Evidence class | All-channel score | Taxon ID | Source URL |
| --- | --- | --- | --- | --- | --- | --- | --- | --- | --- |
| BARD1 | BRCA1 | 0.999 | 0.9 | 0.999895724713243 | Yes | Experiments + databases | 0.999 | 9606 | https://string-db.org/api/tsv/network?identifiers=BRCA1%0DBARD1%0DBRIP1%0DRBBP8%0DABRAXAS1%0DBABAM1%0DUIMC1%0DMRE11%0DRAD50%0DTOPBP1%0DPALB2&species=9606&required_score=0&network_type=functional&caller_identity=codex_brca1_revision |
| BRIP1 | BRCA1 | 0.999 | 0.9 | 0.999895724713243 | Yes | Experiments + databases | 0.999 | 9606 | https://string-db.org/api/tsv/network?identifiers=BRCA1%0DBARD1%0DBRIP1%0DRBBP8%0DABRAXAS1%0DBABAM1%0DUIMC1%0DMRE11%0DRAD50%0DTOPBP1%0DPALB2&species=9606&required_score=0&network_type=functional&caller_identity=codex_brca1_revision |
| RBBP8 | BRCA1 | 0.999 | 0.9 | 0.999895724713243 | Yes | Experiments + databases | 0.999 | 9606 | https://string-db.org/api/tsv/network?identifiers=BRCA1%0DBARD1%0DBRIP1%0DRBBP8%0DABRAXAS1%0DBABAM1%0DUIMC1%0DMRE11%0DRAD50%0DTOPBP1%0DPALB2&species=9606&required_score=0&network_type=functional&caller_identity=codex_brca1_revision |
| MRE11 | RAD50 | 0.998 | 0.9 | 0.9997914494264859 | Yes | Experiments + databases | 0.999 | 9606 | https://string-db.org/api/tsv/network?identifiers=BRCA1%0DBARD1%0DBRIP1%0DRBBP8%0DABRAXAS1%0DBABAM1%0DUIMC1%0DMRE11%0DRAD50%0DTOPBP1%0DPALB2&species=9606&required_score=0&network_type=functional&caller_identity=codex_brca1_revision |
| ABRAXAS1 | BRCA1 | 0.994 | 0.9 | 0.9993743482794578 | Yes | Experiments + databases | 0.999 | 9606 | https://string-db.org/api/tsv/network?identifiers=BRCA1%0DBARD1%0DBRIP1%0DRBBP8%0DABRAXAS1%0DBABAM1%0DUIMC1%0DMRE11%0DRAD50%0DTOPBP1%0DPALB2&species=9606&required_score=0&network_type=functional&caller_identity=codex_brca1_revision |
| ABRAXAS1 | UIMC1 | 0.994 | 0.9 | 0.9993743482794578 | Yes | Experiments + databases | 0.999 | 9606 | https://string-db.org/api/tsv/network?identifiers=BRCA1%0DBARD1%0DBRIP1%0DRBBP8%0DABRAXAS1%0DBABAM1%0DUIMC1%0DMRE11%0DRAD50%0DTOPBP1%0DPALB2&species=9606&required_score=0&network_type=functional&caller_identity=codex_brca1_revision |
| BABAM1 | ABRAXAS1 | 0.994 | 0.9 | 0.9993743482794578 | Yes | Experiments + databases | 0.999 | 9606 | https://string-db.org/api/tsv/network?identifiers=BRCA1%0DBARD1%0DBRIP1%0DRBBP8%0DABRAXAS1%0DBABAM1%0DUIMC1%0DMRE11%0DRAD50%0DTOPBP1%0DPALB2&species=9606&required_score=0&network_type=functional&caller_identity=codex_brca1_revision |
| BABAM1 | BRCA1 | 0.994 | 0.9 | 0.9993743482794578 | Yes | Experiments + databases | 0.999 | 9606 | https://string-db.org/api/tsv/network?identifiers=BRCA1%0DBARD1%0DBRIP1%0DRBBP8%0DABRAXAS1%0DBABAM1%0DUIMC1%0DMRE11%0DRAD50%0DTOPBP1%0DPALB2&species=9606&required_score=0&network_type=functional&caller_identity=codex_brca1_revision |
| BABAM1 | UIMC1 | 0.994 | 0.9 | 0.9993743482794578 | Yes | Experiments + databases | 0.999 | 9606 | https://string-db.org/api/tsv/network?identifiers=BRCA1%0DBARD1%0DBRIP1%0DRBBP8%0DABRAXAS1%0DBABAM1%0DUIMC1%0DMRE11%0DRAD50%0DTOPBP1%0DPALB2&species=9606&required_score=0&network_type=functional&caller_identity=codex_brca1_revision |
| BARD1 | ABRAXAS1 | 0.994 | 0.9 | 0.9993743482794578 | Yes | Experiments + databases | 0.999 | 9606 | https://string-db.org/api/tsv/network?identifiers=BRCA1%0DBARD1%0DBRIP1%0DRBBP8%0DABRAXAS1%0DBABAM1%0DUIMC1%0DMRE11%0DRAD50%0DTOPBP1%0DPALB2&species=9606&required_score=0&network_type=functional&caller_identity=codex_brca1_revision |
| BRCA1 | UIMC1 | 0.994 | 0.9 | 0.9993743482794578 | Yes | Experiments + databases | 0.999 | 9606 | https://string-db.org/api/tsv/network?identifiers=BRCA1%0DBARD1%0DBRIP1%0DRBBP8%0DABRAXAS1%0DBABAM1%0DUIMC1%0DMRE11%0DRAD50%0DTOPBP1%0DPALB2&species=9606&required_score=0&network_type=functional&caller_identity=codex_brca1_revision |
| BRIP1 | TOPBP1 | 0.994 | 0.9 | 0.9993743482794578 | Yes | Experiments + databases | 0.999 | 9606 | https://string-db.org/api/tsv/network?identifiers=BRCA1%0DBARD1%0DBRIP1%0DRBBP8%0DABRAXAS1%0DBABAM1%0DUIMC1%0DMRE11%0DRAD50%0DTOPBP1%0DPALB2&species=9606&required_score=0&network_type=functional&caller_identity=codex_brca1_revision |
| RAD50 | RBBP8 | 0.994 | 0.9 | 0.9993743482794578 | Yes | Experiments + databases | 0.999 | 9606 | https://string-db.org/api/tsv/network?identifiers=BRCA1%0DBARD1%0DBRIP1%0DRBBP8%0DABRAXAS1%0DBABAM1%0DUIMC1%0DMRE11%0DRAD50%0DTOPBP1%0DPALB2&species=9606&required_score=0&network_type=functional&caller_identity=codex_brca1_revision |
| BARD1 | RBBP8 | 0.994 | 0.7 | 0.9981230448383733 | Yes | Experiments + databases | 0.999 | 9606 | https://string-db.org/api/tsv/network?identifiers=BRCA1%0DBARD1%0DBRIP1%0DRBBP8%0DABRAXAS1%0DBABAM1%0DUIMC1%0DMRE11%0DRAD50%0DTOPBP1%0DPALB2&species=9606&required_score=0&network_type=functional&caller_identity=codex_brca1_revision |
| BARD1 | MRE11 | 0.994 | 0.54 | 0.9971220020855057 | Yes | Experiments + databases | 0.999 | 9606 | https://string-db.org/api/tsv/network?identifiers=BRCA1%0DBARD1%0DBRIP1%0DRBBP8%0DABRAXAS1%0DBABAM1%0DUIMC1%0DMRE11%0DRAD50%0DTOPBP1%0DPALB2&species=9606&required_score=0&network_type=functional&caller_identity=codex_brca1_revision |
| BARD1 | RAD50 | 0.994 | 0.54 | 0.9971220020855057 | Yes | Experiments + databases | 0.997 | 9606 | https://string-db.org/api/tsv/network?identifiers=BRCA1%0DBARD1%0DBRIP1%0DRBBP8%0DABRAXAS1%0DBABAM1%0DUIMC1%0DMRE11%0DRAD50%0DTOPBP1%0DPALB2&species=9606&required_score=0&network_type=functional&caller_identity=codex_brca1_revision |
| BRIP1 | BARD1 | 0.994 | 0.54 | 0.9971220020855057 | Yes | Experiments + databases | 0.999 | 9606 | https://string-db.org/api/tsv/network?identifiers=BRCA1%0DBARD1%0DBRIP1%0DRBBP8%0DABRAXAS1%0DBABAM1%0DUIMC1%0DMRE11%0DRAD50%0DTOPBP1%0DPALB2&species=9606&required_score=0&network_type=functional&caller_identity=codex_brca1_revision |
| MRE11 | BRCA1 | 0.994 | 0.54 | 0.9971220020855057 | Yes | Experiments + databases | 0.999 | 9606 | https://string-db.org/api/tsv/network?identifiers=BRCA1%0DBARD1%0DBRIP1%0DRBBP8%0DABRAXAS1%0DBABAM1%0DUIMC1%0DMRE11%0DRAD50%0DTOPBP1%0DPALB2&species=9606&required_score=0&network_type=functional&caller_identity=codex_brca1_revision |
| RAD50 | BRCA1 | 0.994 | 0.54 | 0.9971220020855057 | Yes | Experiments + databases | 0.999 | 9606 | https://string-db.org/api/tsv/network?identifiers=BRCA1%0DBARD1%0DBRIP1%0DRBBP8%0DABRAXAS1%0DBABAM1%0DUIMC1%0DMRE11%0DRAD50%0DTOPBP1%0DPALB2&species=9606&required_score=0&network_type=functional&caller_identity=codex_brca1_revision |
| TOPBP1 | BARD1 | 0.994 | 0.54 | 0.9971220020855057 | Yes | Experiments + databases | 0.998 | 9606 | https://string-db.org/api/tsv/network?identifiers=BRCA1%0DBARD1%0DBRIP1%0DRBBP8%0DABRAXAS1%0DBABAM1%0DUIMC1%0DMRE11%0DRAD50%0DTOPBP1%0DPALB2&species=9606&required_score=0&network_type=functional&caller_identity=codex_brca1_revision |
| TOPBP1 | BRCA1 | 0.994 | 0.54 | 0.9971220020855057 | Yes | Experiments + databases | 0.999 | 9606 | https://string-db.org/api/tsv/network?identifiers=BRCA1%0DBARD1%0DBRIP1%0DRBBP8%0DABRAXAS1%0DBABAM1%0DUIMC1%0DMRE11%0DRAD50%0DTOPBP1%0DPALB2&species=9606&required_score=0&network_type=functional&caller_identity=codex_brca1_revision |
| BRIP1 | MRE11 | 0.994 | 0.5 | 0.9968717413972888 | Yes | Experiments + databases | 0.998 | 9606 | https://string-db.org/api/tsv/network?identifiers=BRCA1%0DBARD1%0DBRIP1%0DRBBP8%0DABRAXAS1%0DBABAM1%0DUIMC1%0DMRE11%0DRAD50%0DTOPBP1%0DPALB2&species=9606&required_score=0&network_type=functional&caller_identity=codex_brca1_revision |
| PALB2 | BRCA1 | 0.944 | 0.9 | 0.9941605839416059 | Yes | Experiments + databases | 0.999 | 9606 | https://string-db.org/api/tsv/network?identifiers=BRCA1%0DBARD1%0DBRIP1%0DRBBP8%0DABRAXAS1%0DBABAM1%0DUIMC1%0DMRE11%0DRAD50%0DTOPBP1%0DPALB2&species=9606&required_score=0&network_type=functional&caller_identity=codex_brca1_revision |
| MRE11 | RBBP8 | 0.855 | 0.9 | 0.9848800834202294 | Yes | Experiments + databases | 0.997 | 9606 | https://string-db.org/api/tsv/network?identifiers=BRCA1%0DBARD1%0DBRIP1%0DRBBP8%0DABRAXAS1%0DBABAM1%0DUIMC1%0DMRE11%0DRAD50%0DTOPBP1%0DPALB2&species=9606&required_score=0&network_type=functional&caller_identity=codex_brca1_revision |
| BARD1 | UIMC1 | 0.477 | 0.9 | 0.9454640250260689 | Yes | Experiments + databases | 0.999 | 9606 | https://string-db.org/api/tsv/network?identifiers=BRCA1%0DBARD1%0DBRIP1%0DRBBP8%0DABRAXAS1%0DBABAM1%0DUIMC1%0DMRE11%0DRAD50%0DTOPBP1%0DPALB2&species=9606&required_score=0&network_type=functional&caller_identity=codex_brca1_revision |
| BRIP1 | PALB2 | 0.292 | 0.9 | 0.9261730969760167 | Yes | Experiments + databases | 0.998 | 9606 | https://string-db.org/api/tsv/network?identifiers=BRCA1%0DBARD1%0DBRIP1%0DRBBP8%0DABRAXAS1%0DBABAM1%0DUIMC1%0DMRE11%0DRAD50%0DTOPBP1%0DPALB2&species=9606&required_score=0&network_type=functional&caller_identity=codex_brca1_revision |
| BARD1 | BABAM1 | 0.063 | 0.9 | 0.9022940563086549 | Yes | Experiments + databases | 0.998 | 9606 | https://string-db.org/api/tsv/network?identifiers=BRCA1%0DBARD1%0DBRIP1%0DRBBP8%0DABRAXAS1%0DBABAM1%0DUIMC1%0DMRE11%0DRAD50%0DTOPBP1%0DPALB2&species=9606&required_score=0&network_type=functional&caller_identity=codex_brca1_revision |
| PALB2 | RBBP8 | 0.398 | 0.577 | 0.7344671532846715 | Yes | Experiments + databases | 0.953 | 9606 | https://string-db.org/api/tsv/network?identifiers=BRCA1%0DBARD1%0DBRIP1%0DRBBP8%0DABRAXAS1%0DBABAM1%0DUIMC1%0DMRE11%0DRAD50%0DTOPBP1%0DPALB2&species=9606&required_score=0&network_type=functional&caller_identity=codex_brca1_revision |
| RBBP8 | UIMC1 | 0.355 | 0.5 | 0.6637122002085505 | Yes | Experiments + databases | 0.958 | 9606 | https://string-db.org/api/tsv/network?identifiers=BRCA1%0DBARD1%0DBRIP1%0DRBBP8%0DABRAXAS1%0DBABAM1%0DUIMC1%0DMRE11%0DRAD50%0DTOPBP1%0DPALB2&species=9606&required_score=0&network_type=functional&caller_identity=codex_brca1_revision |
| BRIP1 | RAD50 | 0.328 | 0.5 | 0.6496350364963503 | Yes | Experiments + databases | 0.723 | 9606 | https://string-db.org/api/tsv/network?identifiers=BRCA1%0DBARD1%0DBRIP1%0DRBBP8%0DABRAXAS1%0DBABAM1%0DUIMC1%0DMRE11%0DRAD50%0DTOPBP1%0DPALB2&species=9606&required_score=0&network_type=functional&caller_identity=codex_brca1_revision |
| MRE11 | UIMC1 | 0.302 | 0.5 | 0.6360792492179353 | Yes | Experiments + databases | 0.746 | 9606 | https://string-db.org/api/tsv/network?identifiers=BRCA1%0DBARD1%0DBRIP1%0DRBBP8%0DABRAXAS1%0DBABAM1%0DUIMC1%0DMRE11%0DRAD50%0DTOPBP1%0DPALB2&species=9606&required_score=0&network_type=functional&caller_identity=codex_brca1_revision |
| ABRAXAS1 | RBBP8 | 0.292 | 0.5 | 0.6308654848800834 | Yes | Experiments + databases | 0.896 | 9606 | https://string-db.org/api/tsv/network?identifiers=BRCA1%0DBARD1%0DBRIP1%0DRBBP8%0DABRAXAS1%0DBABAM1%0DUIMC1%0DMRE11%0DRAD50%0DTOPBP1%0DPALB2&species=9606&required_score=0&network_type=functional&caller_identity=codex_brca1_revision |
| BARD1 | PALB2 | 0.291 | 0.5 | 0.6303441084462982 | Yes | Experiments + databases | 0.987 | 9606 | https://string-db.org/api/tsv/network?identifiers=BRCA1%0DBARD1%0DBRIP1%0DRBBP8%0DABRAXAS1%0DBABAM1%0DUIMC1%0DMRE11%0DRAD50%0DTOPBP1%0DPALB2&species=9606&required_score=0&network_type=functional&caller_identity=codex_brca1_revision |
| TOPBP1 | RBBP8 | 0.276 | 0.5 | 0.6225234619395204 | Yes | Experiments + databases | 0.863 | 9606 | https://string-db.org/api/tsv/network?identifiers=BRCA1%0DBARD1%0DBRIP1%0DRBBP8%0DABRAXAS1%0DBABAM1%0DUIMC1%0DMRE11%0DRAD50%0DTOPBP1%0DPALB2&species=9606&required_score=0&network_type=functional&caller_identity=codex_brca1_revision |
| TOPBP1 | MRE11 | 0.091 | 0.5 | 0.5260688216892596 | Yes | Experiments + databases | 0.733 | 9606 | https://string-db.org/api/tsv/network?identifiers=BRCA1%0DBARD1%0DBRIP1%0DRBBP8%0DABRAXAS1%0DBABAM1%0DUIMC1%0DMRE11%0DRAD50%0DTOPBP1%0DPALB2&species=9606&required_score=0&network_type=functional&caller_identity=codex_brca1_revision |
| BRIP1 | RBBP8 | 0.069 | 0.5 | 0.5145985401459854 | Yes | Experiments + databases | 0.902 | 9606 | https://string-db.org/api/tsv/network?identifiers=BRCA1%0DBARD1%0DBRIP1%0DRBBP8%0DABRAXAS1%0DBABAM1%0DUIMC1%0DMRE11%0DRAD50%0DTOPBP1%0DPALB2&species=9606&required_score=0&network_type=functional&caller_identity=codex_brca1_revision |
| BABAM1 | RAD50 | 0.0 | 0.5 | 0.5 | Yes | Databases only | 0.532 | 9606 | https://string-db.org/api/tsv/network?identifiers=BRCA1%0DBARD1%0DBRIP1%0DRBBP8%0DABRAXAS1%0DBABAM1%0DUIMC1%0DMRE11%0DRAD50%0DTOPBP1%0DPALB2&species=9606&required_score=0&network_type=functional&caller_identity=codex_brca1_revision |
| BABAM1 | RBBP8 | 0.0 | 0.5 | 0.5 | Yes | Databases only | 0.848 | 9606 | https://string-db.org/api/tsv/network?identifiers=BRCA1%0DBARD1%0DBRIP1%0DRBBP8%0DABRAXAS1%0DBABAM1%0DUIMC1%0DMRE11%0DRAD50%0DTOPBP1%0DPALB2&species=9606&required_score=0&network_type=functional&caller_identity=codex_brca1_revision |
| MRE11 | ABRAXAS1 | 0.0 | 0.5 | 0.5 | Yes | Databases only | 0.843 | 9606 | https://string-db.org/api/tsv/network?identifiers=BRCA1%0DBARD1%0DBRIP1%0DRBBP8%0DABRAXAS1%0DBABAM1%0DUIMC1%0DMRE11%0DRAD50%0DTOPBP1%0DPALB2&species=9606&required_score=0&network_type=functional&caller_identity=codex_brca1_revision |
| MRE11 | BABAM1 | 0.0 | 0.5 | 0.5 | Yes | Databases only | 0.689 | 9606 | https://string-db.org/api/tsv/network?identifiers=BRCA1%0DBARD1%0DBRIP1%0DRBBP8%0DABRAXAS1%0DBABAM1%0DUIMC1%0DMRE11%0DRAD50%0DTOPBP1%0DPALB2&species=9606&required_score=0&network_type=functional&caller_identity=codex_brca1_revision |
| PALB2 | MRE11 | 0.0 | 0.5 | 0.5 | Yes | Databases only | 0.87 | 9606 | https://string-db.org/api/tsv/network?identifiers=BRCA1%0DBARD1%0DBRIP1%0DRBBP8%0DABRAXAS1%0DBABAM1%0DUIMC1%0DMRE11%0DRAD50%0DTOPBP1%0DPALB2&species=9606&required_score=0&network_type=functional&caller_identity=codex_brca1_revision |
| PALB2 | RAD50 | 0.0 | 0.5 | 0.5 | Yes | Databases only | 0.665 | 9606 | https://string-db.org/api/tsv/network?identifiers=BRCA1%0DBARD1%0DBRIP1%0DRBBP8%0DABRAXAS1%0DBABAM1%0DUIMC1%0DMRE11%0DRAD50%0DTOPBP1%0DPALB2&species=9606&required_score=0&network_type=functional&caller_identity=codex_brca1_revision |
| RAD50 | ABRAXAS1 | 0.0 | 0.5 | 0.5 | Yes | Databases only | 0.513 | 9606 | https://string-db.org/api/tsv/network?identifiers=BRCA1%0DBARD1%0DBRIP1%0DRBBP8%0DABRAXAS1%0DBABAM1%0DUIMC1%0DMRE11%0DRAD50%0DTOPBP1%0DPALB2&species=9606&required_score=0&network_type=functional&caller_identity=codex_brca1_revision |
| RAD50 | UIMC1 | 0.0 | 0.5 | 0.5 | Yes | Databases only | 0.568 | 9606 | https://string-db.org/api/tsv/network?identifiers=BRCA1%0DBARD1%0DBRIP1%0DRBBP8%0DABRAXAS1%0DBABAM1%0DUIMC1%0DMRE11%0DRAD50%0DTOPBP1%0DPALB2&species=9606&required_score=0&network_type=functional&caller_identity=codex_brca1_revision |
| TOPBP1 | RAD50 | 0.0 | 0.5 | 0.5 | Yes | Databases only | 0.661 | 9606 | https://string-db.org/api/tsv/network?identifiers=BRCA1%0DBARD1%0DBRIP1%0DRBBP8%0DABRAXAS1%0DBABAM1%0DUIMC1%0DMRE11%0DRAD50%0DTOPBP1%0DPALB2&species=9606&required_score=0&network_type=functional&caller_identity=codex_brca1_revision |

**Table S3D. Figure 5A node selection and conservative-network degree**

| Partner rank | Gene | Role | Experimental score | Database score | Restricted selection score | Degree in 45-edge network |
| --- | --- | --- | --- | --- | --- | --- |
| 0 | BRCA1 | Query protein |  |  |  | 10 |
| 1 | BARD1 | First-shell partner | 0.999 | 0.9 | 0.999895724713243 | 10 |
| 2 | BRIP1 | First-shell partner | 0.999 | 0.9 | 0.999895724713243 | 7 |
| 3 | RBBP8 | First-shell partner | 0.999 | 0.9 | 0.999895724713243 | 10 |
| 4 | ABRAXAS1 | First-shell partner | 0.994 | 0.9 | 0.9993743482794578 | 7 |
| 5 | BABAM1 | First-shell partner | 0.994 | 0.9 | 0.9993743482794578 | 7 |
| 6 | UIMC1 | First-shell partner | 0.994 | 0.9 | 0.9993743482794578 | 7 |
| 7 | MRE11 | First-shell partner | 0.994 | 0.54 | 0.9971220020855057 | 10 |
| 8 | RAD50 | First-shell partner | 0.994 | 0.54 | 0.9971220020855057 | 10 |
| 9 | TOPBP1 | First-shell partner | 0.994 | 0.54 | 0.9971220020855057 | 6 |
| 10 | PALB2 | First-shell partner | 0.944 | 0.9 | 0.9941605839416059 | 6 |

**Table S3E. BioGRID v5.0.260 multi-validated human physical partners of BRCA1**

| Evidence rank | Partner | Record count | Unique PubMed count | Unique experimental systems | Experimental systems | Throughput | PubMed IDs | Displayed in Figure 5B | Source URL |
| --- | --- | --- | --- | --- | --- | --- | --- | --- | --- |
| 1 | BARD1 | 118 | 86 | 11 | Affinity Capture-MS; Affinity Capture-Western; Biochemical Activity; Co-crystal Structure; Co-localization; Co-purification; FRET; PCA; Proximity Label-MS; Reconstituted Complex; Two-hybrid | High Throughput; Low Throughput | 8944023; 9342365; 9738006; 9788437; 9798686; 10026184; 10477523; 10635334; 11257228; 11278247; 11301010; 11498787; 11504724; 11526114; 11573085; 11773071; 11925436; 11927591; 12431996; 12438698; 12485996; 12700228; 12732733; 12890688; 12951069; 14636569; 14638690; 14647430; 15159397; 15569676; 15855157; 16391231; 16403807; 16489000; 17525341; 17664283; 18493658; 18936166; 19117993; 19176389; 19369211; 19766185; 19916563; 20060929; 20215511; 20681793; 21901007; 23086937; 23416467; 24085845; 24289923; 24695549; 24981860; 25184681; 25252691; 25634209; 25652403; 25823446; 26350354; 26490435; 26831064; 27239795; 27454289; 27977889; 28262780; 28398198; 28514442; 28948225; 29180510; 29355525; 29367421; 29656893; 29858377; 31270457; 32092106; 32347575; 33589814; 33961781; 34408138; 34591612; 34726323; 35203293; 35487060; 36347866; 37655321; 37797621 | Yes | https://downloads.thebiogrid.org/File/BioGRID/Latest-Release/BIOGRID-MV-Physical-LATEST.tab3.zip |
| 2 | RBBP8 | 55 | 38 | 8 | Affinity Capture-MS; Affinity Capture-Western; Biochemical Activity; Co-localization; Protein-peptide; Proximity Label-MS; Reconstituted Complex; Two-hybrid | High Throughput; Low Throughput | 9738006; 9811458; 10196224; 10764811; 10910365; 11689934; 14578343; 15485915; 16818604; 16843262; 17525340; 17525342; 18171670; 18285836; 19452558; 20351172; 21908405; 22231403; 23416467; 24085845; 25184681; 25252691; 25310973; 25659039; 26387952; 26446986; 27454289; 27940552; 28132842; 28398198; 29513652; 29656893; 30257991; 31636387; 31932421; 34408138; 34591612; 37797621 | Yes | https://downloads.thebiogrid.org/File/BioGRID/Latest-Release/BIOGRID-MV-Physical-LATEST.tab3.zip |
| 3 | BRIP1 | 53 | 36 | 7 | Affinity Capture-MS; Affinity Capture-Western; Co-crystal Structure; Co-localization; Protein-peptide; Proximity Label-MS; Reconstituted Complex | High Throughput; Low Throughput | 11301010; 11877378; 14576433; 14578343; 15125843; 15133502; 15242590; 16391231; 17525340; 17525341; 17525342; 17581638; 17596542; 17664283; 18285836; 18628483; 18717574; 19369211; 19452558; 20159462; 20173781; 20681793; 21240188; 22032289; 23157317; 23530059; 24573678; 25184681; 25252691; 26831064; 27399284; 28398198; 28911102; 29656893; 30257991; 34591612 | Yes | https://downloads.thebiogrid.org/File/BioGRID/Latest-Release/BIOGRID-MV-Physical-LATEST.tab3.zip |
| 4 | UBE2D3 | 35 | 31 | 4 | Affinity Capture-Western; Co-crystal Structure; Reconstituted Complex; Two-hybrid | Low Throughput | 11927591; 12438698; 15367667; 15886201; 15905410; 16543155; 16628214; 16818604; 17283126; 17349954; 17392432; 19712108; 20351172; 21113135; 21531767; 21532592; 21756275; 21965653; 22863316; 23246971; 25355358; 28415827; 28564596; 29180510; 32092106; 32726637; 33589814; 34321665; 37305927; 37633270; 37797621 | Yes | https://downloads.thebiogrid.org/File/BioGRID/Latest-Release/BIOGRID-MV-Physical-LATEST.tab3.zip |
| 5 | UIMC1 | 32 | 21 | 5 | Affinity Capture-MS; Affinity Capture-Western; Co-localization; Proximity Label-MS; Reconstituted Complex | High Throughput; Low Throughput | 17525340; 17525341; 17525342; 17621610; 19261748; 19305427; 19615732; 19766185; 21622030; 24085845; 25184681; 25252691; 26027929; 26446986; 28569838; 29499138; 29656893; 31932421; 34408138; 34591612; 37638744 | Yes | https://downloads.thebiogrid.org/File/BioGRID/Latest-Release/BIOGRID-MV-Physical-LATEST.tab3.zip |
| 6 | PALB2 | 29 | 19 | 4 | Affinity Capture-MS; Affinity Capture-Western; Proximity Label-MS; Reconstituted Complex | High Throughput; Low Throughput | 19268590; 19369211; 19553677; 19584259; 22193777; 22331464; 23038782; 23585894; 24085845; 25016020; 25184681; 25652403; 26649820; 28089683; 28319063; 28398198; 29656893; 30337689; 34408138 | Yes | https://downloads.thebiogrid.org/File/BioGRID/Latest-Release/BIOGRID-MV-Physical-LATEST.tab3.zip |
| 7 | FAM175A | 23 | 18 | 7 | Affinity Capture-MS; Affinity Capture-Western; Co-crystal Structure; Co-localization; Protein-peptide; Proximity Label-MS; Reconstituted Complex | High Throughput; Low Throughput | 17525340; 17643121; 17643122; 19261746; 19261748; 19261749; 19305427; 19766185; 21282113; 22357538; 23416467; 25184681; 26778126; 28398198; 29656893; 30257991; 34591612; 37198153 | Yes | https://downloads.thebiogrid.org/File/BioGRID/Latest-Release/BIOGRID-MV-Physical-LATEST.tab3.zip |
| 8 | ESR1 | 30 | 16 | 5 | Affinity Capture-MS; Affinity Capture-Western; Biochemical Activity; Co-localization; Reconstituted Complex | High Throughput; Low Throughput | 11244506; 11493692; 11782371; 12400015; 15674350; 16061635; 16860316; 17392432; 17505062; 19887647; 20060929; 25499220; 27977889; 29367421; 31527615; 35430604 | Yes | https://downloads.thebiogrid.org/File/BioGRID/Latest-Release/BIOGRID-MV-Physical-LATEST.tab3.zip |
| 9 | RAD51 | 20 | 13 | 8 | Affinity Capture-MS; Affinity Capture-Western; Co-localization; Co-purification; Far Western; Proximity Label-MS; Reconstituted Complex; Two-hybrid | High Throughput; Low Throughput | 9008167; 9774970; 11498787; 14636569; 17302439; 19766185; 24085845; 25499220; 25659039; 28976962; 29656893; 31009828; 34408138 | Yes | https://downloads.thebiogrid.org/File/BioGRID/Latest-Release/BIOGRID-MV-Physical-LATEST.tab3.zip |
| 10 | POLR2A | 15 | 13 | 6 | Affinity Capture-MS; Affinity Capture-Western; Biochemical Activity; Co-localization; Co-purification; Reconstituted Complex | High Throughput; Low Throughput | 9159119; 11504724; 12955082; 14506230; 15886201; 18197258; 22990118; 25184681; 27583302; 29180510; 29513652; 34591612; 34610268 | Yes | https://downloads.thebiogrid.org/File/BioGRID/Latest-Release/BIOGRID-MV-Physical-LATEST.tab3.zip |
| 11 | TP53 | 23 | 13 | 4 | Affinity Capture-MS; Affinity Capture-Western; Co-purification; Reconstituted Complex | Low Throughput | 9482880; 9582019; 9926942; 14636569; 14710355; 15571721; 16677609; 16969499; 18501503; 19509300; 21742769; 26140185; 31073355 | Yes | https://downloads.thebiogrid.org/File/BioGRID/Latest-Release/BIOGRID-MV-Physical-LATEST.tab3.zip |
| 12 | H2AFX | 15 | 13 | 4 | Affinity Capture-Western; Biochemical Activity; Co-localization; Reconstituted Complex | Low Throughput | 10959836; 11927591; 12485996; 18344987; 19766185; 19805520; 20681793; 26095369; 26121674; 29499138; 31501894; 34321665; 36906594 | Yes | https://downloads.thebiogrid.org/File/BioGRID/Latest-Release/BIOGRID-MV-Physical-LATEST.tab3.zip |
| 13 | BRCA2 | 15 | 12 | 6 | Affinity Capture-MS; Affinity Capture-Western; Co-purification; Proximity Label-MS; Reconstituted Complex; Two-hybrid | High Throughput; Low Throughput | 9774970; 11477095; 14499622; 14636569; 17664283; 19369211; 21135251; 24085845; 25184681; 25652403; 29656893; 34408138 | Yes | https://downloads.thebiogrid.org/File/BioGRID/Latest-Release/BIOGRID-MV-Physical-LATEST.tab3.zip |
| 14 | UBE2D1 | 15 | 12 | 3 | Co-crystal Structure; Reconstituted Complex; Two-hybrid | Low Throughput | 11278247; 12485996; 12732733; 12887909; 12890688; 14636569; 14638690; 16403807; 19712108; 19916563; 24507701; 27312108 | Yes | https://downloads.thebiogrid.org/File/BioGRID/Latest-Release/BIOGRID-MV-Physical-LATEST.tab3.zip |
| 15 | MRE11A | 13 | 11 | 6 | Affinity Capture-MS; Affinity Capture-Western; Co-localization; Co-purification; Proximity Label-MS; Reconstituted Complex | High Throughput; Low Throughput | 10426999; 10783165; 11353843; 11504724; 16391231; 17349954; 18763866; 23530059; 24413181; 25184681; 29656893 | Yes | https://downloads.thebiogrid.org/File/BioGRID/Latest-Release/BIOGRID-MV-Physical-LATEST.tab3.zip |
| 16 | NBN | 12 | 11 | 5 | Affinity Capture-MS; Affinity Capture-Western; Co-localization; Co-purification; Proximity Label-MS | High Throughput; Low Throughput | 10426999; 10783165; 11504724; 12124328; 18171670; 18763866; 19244116; 25184681; 25659039; 25939603; 29656893 | Yes | https://downloads.thebiogrid.org/File/BioGRID/Latest-Release/BIOGRID-MV-Physical-LATEST.tab3.zip |
| 17 | RAD50 | 14 | 10 | 7 | Affinity Capture-MS; Affinity Capture-Western; Co-localization; Co-purification; Proximity Label-MS; Reconstituted Complex; Two-hybrid | High Throughput; Low Throughput | 10426999; 10783165; 11504724; 12124328; 16391231; 18171670; 18763866; 25084169; 25184681; 29656893 | Yes | https://downloads.thebiogrid.org/File/BioGRID/Latest-Release/BIOGRID-MV-Physical-LATEST.tab3.zip |
| 18 | ATM | 12 | 9 | 6 | Affinity Capture-Western; Biochemical Activity; Co-purification; Protein-peptide; Proximity Label-MS; Reconstituted Complex | High Throughput; Low Throughput | 10550055; 10608806; 10783165; 10866324; 11114888; 15713674; 17478428; 18344987; 29656893 | Yes | https://downloads.thebiogrid.org/File/BioGRID/Latest-Release/BIOGRID-MV-Physical-LATEST.tab3.zip |
| 19 | HIST1H2AB | 10 | 9 | 2 | Biochemical Activity; Co-crystal Structure | Low Throughput | 25131202; 25355358; 29367421; 32092106; 33589814; 34321665; 37305927; 37797621; 37823591 | Yes | https://downloads.thebiogrid.org/File/BioGRID/Latest-Release/BIOGRID-MV-Physical-LATEST.tab3.zip |
| 20 | ACACA | 12 | 8 | 5 | Affinity Capture-MS; Affinity Capture-Western; Co-crystal Structure; Protein-peptide; Reconstituted Complex | Low Throughput | 12360400; 16326698; 16698035; 18452305; 19061860; 24565757; 29899443; 30257991 | Yes | https://downloads.thebiogrid.org/File/BioGRID/Latest-Release/BIOGRID-MV-Physical-LATEST.tab3.zip |
